# Cross‐species comparison illuminates the importance of iron homeostasis for splenic anti‐immunosenescence

**DOI:** 10.1111/acel.13982

**Published:** 2023-09-08

**Authors:** Ziqing He, Weiya He, Chuanxia Hu, Jiayu Liao, Wenjun Deng, Haijian Sun, Qingpei Huang, Weilue Chen, Libiao Zhang, Meiling Liu, Ji Dong

**Affiliations:** ^1^ GMU‐GIBH Joint School of Life Sciences, The Guangdong‐Hong Kong‐Macau Joint Laboratory for Cell Fate Regulation and Diseases, Guangzhou National Laboratory Guangzhou Medical University Guangzhou China; ^2^ Faculty of Health Sciences University of Macau Macau China; ^3^ Bioland Laboratory (Guangzhou Regenerative Medicine and Health Guangdong Laboratory) Guangzhou China; ^4^ Guangdong Key Laboratory of Animal Conservation and Resource Utilization, Guangdong Public Laboratory of Wild Animal Conservation and Utilization Institute of Zoology, Guangdong Academy of Sciences Guangzhou China

**Keywords:** cross‐species comparison, immunosenescence, iron homeostasis, *PCBP1*, single‐cell transcriptomics, splenic immune cells

## Abstract

Although immunosenescence may result in increased morbidity and mortality, many mammals have evolved effective immune coping strategies to extend their lifespans. Thus, the immune systems of long‐lived mammals present unique models to study healthy longevity. To identify the molecular clues of anti‐immunosenescence, we first built high‐quality reference genome for a long‐lived myotis bat, and then compared three long‐lived mammals (i.e., bat, naked mole rat, and human) versus the short‐lived mammal, mouse, in splenic immune cells at single‐cell resolution. A close relationship between B:T cell ratio and immunosenescence was detected, as B:T cell ratio was much higher in mouse than long‐lived mammals and significantly increased during aging. Importantly, we identified several iron‐related genes that could resist immunosenescence changes, especially the iron chaperon, *PCBP1*, which was upregulated in long‐lived mammals but dramatically downregulated during aging in all splenic immune cell types. Supportively, immune cells of mouse spleens contained more free iron than those of bat spleens, suggesting higher level of ROS‐induced damage in mouse. *PCBP1* downregulation during aging was also detected in hepatic but not pulmonary immune cells, which is consistent with the crucial roles of spleen and liver in organismal iron recycling. Furthermore, *PCBP1* perturbation in immune cell lines would result in cellular iron dyshomeostasis and senescence. Finally, we identified two transcription factors that could regulate *PCBP1* during aging. Together, our findings highlight the importance of iron homeostasis in splenic anti‐immunosenescence, and provide unique insight for improving human healthspan.

## Graphical abstract

To identify molecular clues of anti‐immunosenescence, we compare three long‐lived mammals versus the short‐lived mammal, mouse, in splenic immune cells at single‐cell resolution. We detect higher splenic B:T cell ratio and higher free iron level in mice, while higher expression levels of iron homeostasis‐related genes in long‐lived species. Our findings highlight the importance of iron homeostasis in splenic anti‐immunosenescence, and provide unique insight for improving human healthspan.

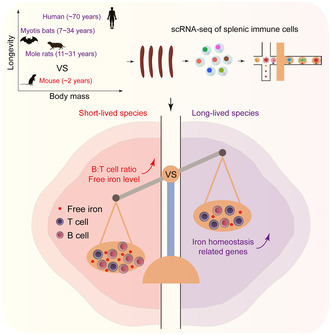

**Abbreviations**
BUSCO: benchmarking universal single‐copy orthologs
Cistrome DB: Ccistrome Ddata Bbrowser
DEGs: differentially expressed genes
GO: Ggene Oontology
Hi‐C sequencing: high‐throughput chromosome conformation capture sequencing
KD: knockdown
KEGG: Kyoto Eencyclopedia of Ggenes and Ggenomes Ddatabase
LIP: labile iron pool
Mb: megabase
N50: length of the shortest contig for which longer and equal length contigs cover at least 50 % of the assembly
NC: negative control
NES: normalized enrichment score
NK cells: natural killer cells
NMRs: naked mole rats
ROS: reactive oxygen species
scATAC‐seq: single‐cell assay for transposase‐accessible chromatin sequencing
scRNA‐seq: single‐cell RNA sequencing
SD: standard deviation
shRNA: short hairpin RNA
siRNA: small interfering RNA
TPM: transcripts per kilobase of exon model per million mapped reads
UMAP: uniform manifold approximation and projection

## 1 INTRODUCTION

The immune system protects the organism by fighting infection, eliminating cancerous cells, and regulating tissue repair, etc., which is critical for the organismal homeostasis and longevity (Borgoni et al., 2021). The gradual deterioration of the immune system during aging, known as immunosenescence, may result in increased morbidity and mortality in animals (Barbé‐Tuana et al., 2020). Although the importance of immunosenescence has been recognized for a long time, its underlying mechanisms remain elusive.

Lifespan varies over 100‐fold across mammals, and usually positively correlates with their body mass (Kowalczyk et al., 2020). For example, the maximum lifespans of mouse and whale are reported to be ~4 and ~211 years, respectively (Keane et al., 2015). During evolution, many mammals have evolved specific adaptations of immune systems, which significantly contribute to their extreme longevity. After size correction, myotis bats, naked mole rats (NMRs), and human possess much more exceptional longevity than mice (Tollis et al., 2020). Thus, the immune systems of these long‐lived mammals present unique models to study the mechanisms of anti‐immunosenescence and healthy longevity.

As the largest secondary lymphoid organ of the body, the spleen plays a critical role in hematopoiesis and blood filtration (Lewis et al., 2019). Aged, infected, or dysfunctional red blood cells are removed from the circulation by splenic red pulp macrophages, and their iron is reclaimed for systemic use (Jebb et al., 2020). Besides, the spleen contains nearly one‐fourth of all lymphocytes necessary for innate and adaptive immune responses (Bronte & Pittet, 2013). In this study, we make cross‐species comparison of splenic immune cells between three long‐lived mammals (i.e., myotis bat (*Myotis ricketti*), NMR, and human) and the short‐lived mammal, mouse, at single‐cell resolution, and we aim to (i) uncover the similarities and differences in the cellular diversity and gene expression pattern of immune cells across these species; (ii) identify marker genes that are more highly expressed in long‐lived species, which may have anti‐immunosenescence potentials; (iii) and finally explore their roles in anti‐immunosenescence. In summary, the systematical comparison of splenic immune cells between long‐lived versus short‐lived mammals will provide novel insights in understanding the molecular mechanisms of anti‐immunosenescence and healthy longevity.

## 2 METHODS

### 2.1 Animal models

Myotis bats were sampled in accordance with the permits and ethical guidelines (License No. GIZ20220302) issued by Institute of Zoology, Guangdong Academy of Science. Two male and two female myotis bats were used for single‐cell RNA sequencing (scRNA‐seq), and four male myotis bats were used for LIP assay. Young (8–10 weeks old, male) and aged (20 months old, male) wild‐type C57BL/6J mice were purchased from Casgene Biotech CO., Ltd. All mice were housed under standard environment and provided with clean water and food ad libitum. The Administrative Panel on Laboratory Animal Care at the Guangzhou Laboratory approved all the experimental procedures in this study.

Maximum longevity (years) and adult body mass (g) of mammals were downloaded from the AnAge database (Tacutu et al., 2012). The relationships of body mass and longevity is plotted by fitting a linear regression in R v4.2.1 (https://www.R‐project.org/).

### 2.2 The isolation of splenic immune cells

Animals were anesthetized by intraperitoneal injection of isoflurane, then the spleens were harvested and immediately transferred into 5 mL iced RPMI 1640 with 10% FBS, and then cut into small pieces (~0.2 cm^2^) with a scissor. Splenic immune cells were released by grinding the spleen fragments through a 100‐μm cell strainer with a syringe plunger, or pipetting gently with a Pasteur pipette. Cells were washed with RPMI 1640 with 10% FBS and centrifuged at 450 g for 5 min at 4°C, then resuspended with 3 mL ACK Lysing Buffer (Gibco, A1049201) for 5 min at room temperature to remove red blood cells. Cells were then washed twice and resuspended in PBS with 0.1% BSA, passed through a 40‐μm filter, counted and checked viability using trypan blue before use. The cells and the wash buffer should be kept on ice during the whole procedure to preserve cell viability.

### 2.3 scRNA‐seq and bulk RNA‐seq

Single‐cell libraries of bat splenic immune cells were generated with 10X genomic platform using the Chromium Next GEM single Cell 3’ Reagent Kits v3.1 following the manufacturer's instruction. For bulk RNA‐seq of different cell lines, total RNA was extracted using RNeasy Mini kit (QIAGEN, 74106), libraries were prepared using VAHTS RNA‐seq V3 Library Prep Kit for Illumina (Vazyme, NR611) following the manufacturer's instruction. Quality of both scRNA‐seq and bulk RNA‐seq libraries was evaluated by the Fragment Analyzer (Advanced Analytical). Libraries were sequenced on an illumine NovaSeq6000 in the PE150 mode.

### 2.4 Genome assembly and genome annotation

We performed Illumina sequencing and Nanopore sequencing using the DNA from muscle tissues of adult male bats. First, Illumina data were used to estimate the genome size and heterozygosity using GenomeScope (Vurture et al., 2017) and K‐mer was set as 21. Second, NextDenovo (https://github.com/Nextomics/NextDenovo, NextDenovo v.2.3.1) was used to assemble the contigs with different seed depth, including 45X, 50X, and 55X. 50X seed depth was used based on the N50 length and total length of the assembly results. Third, NextPolish (https://github.com/Nextomics/NextPolish, NextPolish v.1.3.0) was used to polish the contigs by using Illumina data with default parameters. Fourth, Juicer v.1.6 (Durand et al., 2016) and 3D‐DNA v.180922 (Dudchenko et al., 2017) were used to link the polished contigs with Hi‐C data. Finally, a genome‐wide Hi‐C heatmap was generated by JuicerBox v.1.11.08 (Robinson et al., 2018) for manual correction. The final genome fasta was regenerated by 3D‐DNA with the corrected assembly files. Genome assembly quality and gene integrity were evaluated by using Benchmarking Universal single‐copy orthologs (BUSCO v4.1.4) (Manni et al., 2021) with mammalian (odb10) dataset, including 9226 conserved genes.

We performed Illumina short‐read sequencing (Illumina RNA‐seq) and Oxford Nanopore technologies long‐read sequencing (ONT RNA‐seq) on 13 tissues of adult male bats for genome annotation. These tissues included heart, liver, spleen, lung, kidney, intestine, cochlea, stomach, pancreas, muscle, brain, eye, and testis. Meanwhile, Illumina RNA‐seq data from NCBI, and well‐annotated protein sequence from human, mouse, and other bats were also collected for genome annotation. Briefly, genome annotation mainly included three steps: ab initio gene prediction, protein homology‐based and transcriptome‐based prediction.

For transcriptome‐based prediction, Illumina short reads data were mapped to the genome assembly using HISAT2 v2.2.1 (Kim et al., 2019), the alignments results were assembled into gene structures using StringTie v2.1.4 (Kovaka et al., 2019), indicated as StringTie‐evidence. Illumina RNA‐seq data were also used to perform genome‐guided assembly and de novo assembly by using Trinity v2.11.0 (Grabherr et al., 2011). The results of de novo RNA‐seq assembly and genome‐guided RNA‐seq assembly were denoted as Trinity‐evidence. Pychopper v.2.4.0 (https://github.com/nanoporetech/pychopper) was used to trim ONT long reads and identify the full‐length cDNA reads. The identified full‐length reads were mapped to genome assembly using minimap2 v2.24 (Li, 2018) in “map‐ont” mode, and transcripts were assembled using StringTie. Transcriptome‐based gene structures were modeled using PASApipeline v2.5.2 (PASA‐evidence) based on above results. ORFs were extracted from gene structure based on PASA‐evidence and were used for training data for ab initio gene prediction.

For ab initio gene prediction, repeat sequences in genome were first identified by RepeatModeler v2.0.1 (Flynn et al., 2020) and the results were used to build species‐specific repeat library. And then the repeat library was integrated together with RepBase v20181026 to mask genome assembly by RepeatMasker v4.0.8 (http://www.repeatmasker.org). SNAP v2013‐11‐ 529 and AUGUSTUS v3.2.3 were implemented to MAKER v2.31.11 (Holt & Yandell, 2011) genome annotation pipelines to perform ab initio gene prediction.

For protein homology‐based prediction, well‐annotated proteins from 10 UniProt v20210405 and bat proteins from NCBI, and aligned the proteins to genome assembly with GenomeThreader 1.7.1 (gth‐evidence) were collected. Then we used EvidenceModeler v1.1.1 to integrate the above collected evidences into consensus gene models. The weights we used on the integration step were 12 for ONT‐evidence, 11 for PASA‐evidence, 10 for StringTie‐evidence, 9 for Trinity‐evidence, 5 for gth‐evidence, and 2 for marker‐evidence. Finally, alternative splicing models and UTRs were added to EVM‐models by using PASApipeline in “genome annotation” mode. The downstream inspection and function annotation were based on the resulted PASA‐models.

TransDecoder v5.5.0 (http://transdecoder.github.io) were used to predict the putative peptides for PASA‐model and the results were aligned against to the Uniprot database by using blastp v2.10.0+ with E‐value below 1e‐6. Peptides with no hits and length below 100 amino acids were removed from the PASA‐models. BUSCO v4.1.4 with mammalian (odb10) dataset was used to evaluate the completeness of annotation (Manni et al., 2021).

### 2.5 Phylogenetic analysis of species tree

OMA standalone v. 2.4.2 was used to identify the orthologous genes of target species (Altenhoff et al., 2019). First, we exported the precomputed all‐against‐all genomes from OMA BROWSER (https://omabrowser.org/oma/export/), including *Canis lupus familiaris*, *Felis catus*, *Bos taurus*, *Sus scrofa*, *Equus caballus*, *Homo sapiens*, *Macaca mulatta*, *Mus musculus*, *Rattus norvegicus*, *Pteropus vampyrus*, and *Myotis lucifugus*. Other bat species proteomes, such as *Phyllostomus discolor* (GCF_004126475.2), *Pipistrellus kuhlii* (GCF_014108245.1), *Rhinolophus ferrumequinum* (GCF_004115265.1), *Rousettus aegyptiacus* (GCF_014176215.1), *Molossus molossus* (GCF_014108415.1), and *Myotis myotis* (GCF_014108235.1) were downloaded from NCBI. The proteomes of *M. ricketti* was generated by TransDecoder v.5.5.0 (https://github.com/TransDecoder/TransDecoder) based on the genomes we got. Second, we filtered the orthologous groups using the filter_groups.py file (available at https://zenodo.org/record/3786201) by setting the number of species to 16. Third, MAFFT v7.48 (Katoh, 2002) was used to align the filtered orthologous groups and all alignments were concatenated into one single multiple sequences alignments using the concate_alignments.py script (available at https://zenodo.org/record/3786201). Finally, the phylogenetic species tree was built using IQTREE v. 2.0.3 with LG model and was visualized by FigTree v1.4.4 (http://tree.bio.ed.ac.uk/software/figtree/). The node supports of the phylogenetic tree was obtained by 1000 replicated of bootstrapping. *Mus musculus* was used as outgroup.

### 2.6 Cell line culture, siRNA and shRNA knockdown, and RT‐qPCR

The mouse macrophage cell line Raw264.7 and the microglial cell line EOC20 were grew in DMEM (high glucose) supplemented with 10% FBS and 1% penicillin–streptomycin. The mouse B cell line WEHI231 and human T cell line Jurkat were grew in RPMI 1640 supplemented with 10% FBS and 1% penicillin–streptomycin.

Transfection of siRNAs was preformed using Lipofectamine RNAiMAX Reagent (Invitrogen, 13778150) according to the manufacturer's instruction, cell samples were collected 48 h after transfection. *Pcbp1* knockdown in Raw264.7, WEHI231 and Jurkat cell lines were established by transduction of shRNA lentivirus. Non‐target control (NC) and *Pcbp1* shRNA sequence were cloned into pLVX‐shRNA2‐ZsGreen1‐Puro vector. Package of lentivirus was performed by transfecting shRNA vectors into 293T cells using Lipofectamine 3000 Reagent (L3000015, Invitrogen), 48 h after transfection, the supernatants containing lentivirus were collected and concentrated using 5 × Lentivirus Concentration Reagent (C103, GenStar), then stored in aliquots at −80°C before use.

For RT‐qPCR, total RNA from all cell lines, mouse and bat splenic immune cells were extracted using EZ‐press RNA Purification Kit (EZBioscience, B0004D). All cDNA was synthesized using HiScript III RT SuperMix for qPCR kit (Vazyme, R323). qPCR was performed using ChamQ SYBR qPCR Master Mix (Vazyme, Q311) on Quantagene q225 system (KUBO, China). All sequence of siRNA and RT‐qPCR primers were listed in Table S10.

### 2.7 LIP, non‐heme iron assay, cell viability, and phagocytosis experiment

We first compared between bats and mice, where four male myotis bats and four mice were used for LIP assay. Then we compared between young and aged mice, where young (2 months old, male) and aged (20 months old, male) mice were fed with standard diet and high iron diet (8.3 g carbonyl iron/kg, Xietong Biotech) for 4 weeks, respectively. LIP of the splenic immune cells was evaluated as described previously. For each sample, 1 × 10^6^ cells/ml in PBS with 0.1% BSA were incubated with 50 nM calcein‐AM (Beyotime, C2012) for 15 min at 37°C in the dark for free iron chelation. Cells were immediately analyzed on a flow cytometer (Agilent Novocyte Advanteon), the mean florescence intensity (MFI) was measured at the FITC channel. Followed by incubation of 100 μM SIH (Blpbio, 495841) at room temperature for 30 min, cells were analyzed again. Cellular LIP level was estimated as the MFI difference (△F).

The NC and *Pcbp1* knockdown immune cell lines were treated with different concentration of ferric citrate (HY‐N1428C, MedChemExpress) for 4 days. Non‐heme iron concentrations were determined using the Iron assay kit (Abcam, ab83366) following the manufacturer's instruction. Ferric and ferrous iron levels were calculated from the standard curve and normalized to the protein content for each sample using BCA Protein Assay Kit (C503021, Sangon Biotech).

For cell viability assay, cells from both NC and *Pcbp1* shRNA knockdown groups were plated in 96‐well‐plate, 2500 cells/well. Cell viability was assayed using Cellcounting‐Lite 2.0 Luminescent Cell Viability Assay (Vazyme, DD1101) following the manufacturer's instruction, cell proliferation ratio was calculated from Day 1 to Day 4.

For phagocytosis assay, NC and *Pcbp1* knockdown Raw264.7 cells were incubated in 10 ug/ml Zymosan A (S. cerevisiae) BioParticles conjugated with Alexa Fluor 594 (Z23374, Invitrogen) for 3 h. After thorough washing, cells were visualized under a fluorescent microscope, then analyzed on a flow cytometer (Agilent Novocyte Advanteon) to detect the percentage of phagocytic cells.

### 2.8 scRNA‐seq analysis

scRNA‐seq fastq files were demultiplexed to their respective barcodes using the 10 Genomics Cell Ranger mkfastq utility. Unique molecular identifier (UMI) counts were generated for each barcode using the Cell Ranger (v5.0.1) count utility. The bat reference genome was generated by ourselves.

Count data were processed with the Seurat package (v4.1.0). For quality control, genes that were expressed in fewer than three cells and cells that expressed fewer than 100 genes were excluded from analysis. Integration was performed using canonical correlation analysis (CCA), essentially according to the Standard Workflow protocol provided by the authors. The filtered gene‐barcode matrix was analyzed by PCA. Then Uniform Manifold Approximation and Projection (UMAP) was performed on the top 30 principal components for visualizing the cells. Meanwhile, graph‐based clustering was performed on the PCA‐reduced data for clustering analysis with Seurat. Clusters were identified by the expression of known cell‐type markers: B cells (*CD79A* and *CD79B*); T cells (*CD3E* and *CD3G*); macrophages (*CD68* and *CD86*); neutrophils (*S100A8* and *IL1R2*); NK cells, (*KLRC1* and *NKG7*); *Ltf*‐high neutrophils: (*CAMP* and *LTF*), and erythroid cells (*AHSP* and *TFRC*). To identify differentially expressed genes between cell types across species, we used a negative binomial model as implemented in the Seurat ‘FindMarkers’ function, comparing each individual cell type to all other cells within the major cell type cluster (B cells, T cells, APCs, NK cell, and neutrophils). Genes were considered differentially expressed if the adjusted *p* value was lower than 0.05. The single‐cell dataset of young and aged mice spleen was compared to get the upregulated genes in the young mouse splenic immune cells. Then we defined the upregulated genes in the long‐lived species and in the young mice as candidate anti‐immunosenescene genes.

### 2.9 Bulk RNA‐seq analysis

Reads were aligned to mm10 mouse genome (GENCODE vM23/Ensembl 98) using STAR (Dobin et al., 2013), and reads falling on exons of genes were counted using RSEM. Differential expressed genes between different conditions was determined using DESeq2 (Love et al., 2014). Differentially expressed exons between different conditions was done using DEXSeq (Anders et al., 2012). The anti‐immunosenescence scores and immunoscenescence scores of siRNA knockdown groups were calculated by AuCell (Aibar et al., 2017) using the genes upregulated in long‐lived species and genes downregulated in long‐lived species, respectively. Public bulk RNA‐seq datasets of human splenic immune cells of different ages were downloaded from Encode. We downloaded the genes expression files from ENCSR265NZF, ENCSR700QVJ, ENCSR663IOE, ENCSR510PSL, ENCSR910QOX, ENCSR330UMQ, ENCSR194HVU, ENCSR900SGE, and ENCSR106SZN experiment.

### 2.10 Processing and analysis of public scATAC‐seq datasets

scATAC‐seq of CD45^+^ cells datasets from the spleen from young and aged male mice were downloaded from https://www.synapse.org repository (syn22255433). Fastq files were aligned to the mouse reference (mm10) and “cellranger‐atac count” function (cellranger‐atac, v2.0.0) was used to generate single‐cell accessibility counts.

All downstream scATAC‐seq analysis was conducted by using ArchR (Granja et al., 2021). Briefly, the resulting fragment files were used to create an Arrow file and create an ArchRProject after filtering the doublets. Dimensionality reduction and clustering analysis were performed by using the “addIterativeLSI” and “addClusters” function. UMAP was ran by using the “addUMAP” function and visualized by “plotEmbedding” function. Gene activity scores were generated based on the Gene Score model and clusters were annotated based on gene accessibility score of known marker genes. Pseudo‐bulk replicates were made using the “addGroupCoverages” function. Motif annotations were added using “addMotifAnnotations” function. Single‐cell RNA data of young and aged mouse spleen from Mogilenko et al. (Mogilenko et al., 2021) were integrated analysis by “addGeneIntegrationMatrix” function. Peak‐to‐gene links were identified by using “addPeak2GeneLinks” function to find correlations between peak accessibility and gene expression.

### 2.11 Processing and analysis of public scRNA‐seq datasets

Single‐cell datasets from human liver of different ages were download from Liver Cell Atlas (https://livercellatlas.org/download.php) (Guilliams et al., 2022). We used “AverageExpression” function from Seurat (v4.1.0) to check the average expression of *PCBP1* in human liver macrophages at different ages. Single‐cell data of different tissues of mouse were obtained from *Tabula Muris Senis* (The Tabula Muris Consortium et al. 2018). We checked the expression pattern of *Pcbp1* in different tissues at different ages based on data from the microfluidic droplet approach.

### 2.12 Enrichment analysis and TF analysis

Metascape was used to perform Gene ontology (GO) biological process enrichment analysis (Zhou et al., 2019). The enrichKEGG function from the clusterProfiler package (v4.4.4) (Wu et al., 2021) was used to perform KEGG pathway enrichment analysis.

For TF analysis, we used two methods to identify the candidate TFs that might regulate *Pcbp1*. First, Cistrome Toolkit (dbtoolkit.cistrome.org) of existing genome‐wide ChIP‐seq data were used to probe which transcription factors might regulate *Pcbp1* in mouse. The half‐decay distance to transcription start site was set as 10 kb. Second, SCENIC was used to identify regulons based on genes co‐expression and DNA‐motif analysis (Aibar et al., 2017). The single‐cell data of mouse spleen were used to run SCENIC. The candidate transcription factors that might regulate *Pcbp1* were collected. Finally, the top 20 transcription factors found in Cistrome Toolkit intersected with all TFs found in SCENIC, and the resulting transcription factors were regarding as the final candidate transcription factors for *Pcbp1*.

## 3 RESULTS

### 3.1 Splenic immune cell atlas of long‐lived and short‐lived mammals

**FIGURE 1:**
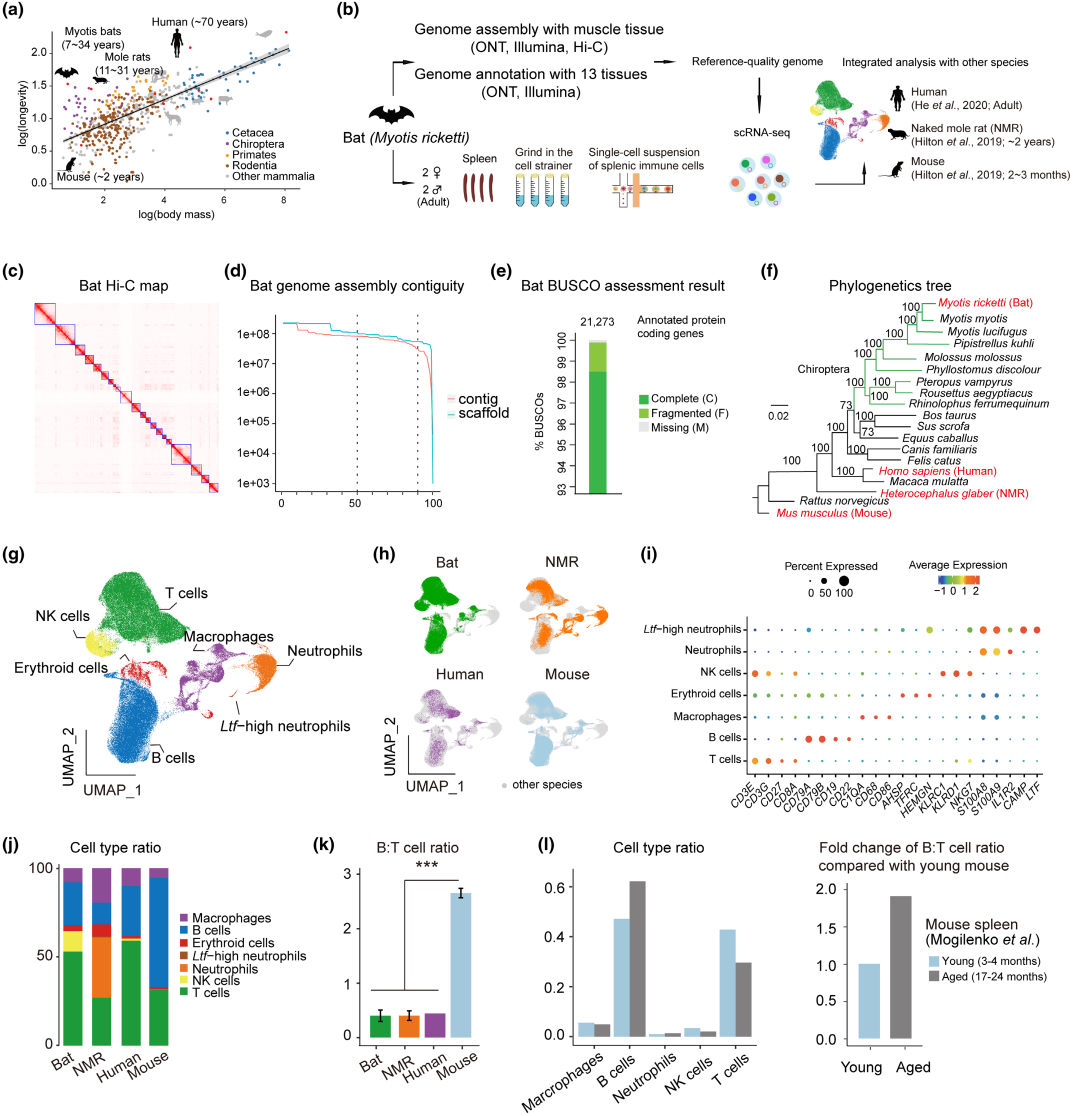
Cross‐species expression atlas of splenic immune cells. (a) Correlation between lifespan and body mass across various mammalian species. Data were downloaded from the AnAge database. (b) Schematic of experimental design showing the workflow of this study. Single‐cell datasets of human, NMR, and mouse are acquired from previous studies. (c) Hi‐C map of myotis bat. (d) Genome assembly contiguity of myotis bat. (e) BUSCO assessment showing completeness of the gene annotation of myotis bat. (f) Phylogenetics tree of myotis bat among mammals. (g and h) Integrated (g) and separated (h) UMAP visualization of splenic immune cells isolated from myotis bat, NMR, human, and mouse. (i) Dotplots showing marker genes expression of each cell type. (j) Bar charts showing the cell type composition of splenic immune cells across four species. (k) B:T cell ratio in splenic immune cells across the four species. Data are mean values ± SD. *** indicates *p* < 0.001 (Student's *t* test). (l) Proportions of five immune cell types (left) and B:T cell ratio (right) in splenic immune cells compared between young and aged mouse.

To systematically compare the similarities and differences of splenic immune cells between long‐lived versus short‐lived mammals, we combined scRNA‐seq datasets sampled from myotis bats, NMRs, human, and mice (Figure 1a,b; Table S1). Among these datasets, bat splenic immune cells were newly generated from two adult male and two adult female myotis bats using 10X Genomics technique, while NMR, mouse, and human datasets were obtained from previous studies (He et al., 2020; Hilton et al., 2019).

Due to the lack of *M. ricketti* reference genome, we newly generated the bat genome by the following workflow. First, we extracted genomic DNA from muscle tissues of adult male bats and performed de novo assembly using third‐generation long reads sequencing on Oxford Nanopore, then the contigs were corrected by Illumina next‐generation sequencing (Figures 1b and S1a). Second, we preformed Hi‐C sequencing to determine the chromosome borders (Figures 1c and S1b). Third, we used 13 tissues of adult male bats to generate transcriptome data by both third‐generation long reads and second‐generation short reads for genome annotation (Figure S1c). Finally, we obtained high‐quality reference genome with the N50 values of 81.7 Mb for contigs and 107.7 Mb for scaffolds, and 21,273 protein‐coding genes were annotated with >99.8% completeness (Figure 1d,e; Table S2). Thus, the quality of this genome was comparable to the best reference‐quality genomes for bats so far (Jebb et al., 2020). We resolved the phylogenetic tree using our newly generated bat genome with reference‐quality genomes of other bat species, and confirmed the right phylogenetic position of *M. ricketti* in Chiroptera (Figure 1f).

Based on our high‐quality bat reference genome, we accurately quantified the gene expression of bat scRNA‐seq dataset (Figure S1d), and integrated all single cells of these four species. After stringent quality control and batch effect correction by Harmony (Korsunsky et al., 2019), all integrated single cells could be grouped into seven major clusters using graph‐based algorithm in Seurat (Figures 1g,h and S1e) (Hao et al., 2021). Based on classic marker genes, we annotated these clusters as B cells (*CD79A* and *CD79B*), T cells (*CD3E* and *CD3G*), macrophages (*CD68* and *CD86*), neutrophils (*S100A8* and *IL1R2*), NK cells (*KLRC1* and *NKG7*), *Ltf*‐high neutrophils (*CAMP* and *LTF*), and erythroid cells (*AHSP and TFRC*) (Figures 1i and S1f).

### 3.2 High splenic B:T cell ratio implies immunosenescence

As shown in Figures 1j and S1g, the cellular composition of splenic immune cells varied significantly among these four species. The NK cell proportion of bat was significantly higher than those of other three species. NMR had more macrophages and neutrophils but no NK cells, which was consistent with previous results (Hilton et al., 2019). Of note, compared with long‐lived species, mouse had more B cells but less T cells, namely a much higher B:T cell ratio, while the B:T cell ratio was comparable among other three long‐lived species (Figure 1k). As previous studies have reported increased B:T cell ratio under pathological conditions (Becklund et al., 2016), we wondered whether the high B:T cell ratio exerted influence on aging. We compared the proportions of five immune cell types and the B:T cell ratio between young and aged mouse splenic immune cells using another published scRNA‐seq dataset (Mogilenko et al., 2021). Indeed, increased B cells and decreased T cells were detected during aging, resulting in a higher splenic B:T cell ratio in aged mice (Figure 1l). Therefore, splenic B:T cell ratio is higher in mouse than long‐lived mammals and increases during aging, implying its correlation with immunosenescence.

To further reveal the heterogeneity of B cells and T cells, we re‐clustered them and identified their respective subpopulations based on expression patterns of typical genes. T cells were subdivided into nine subpopulations, namely naïve CD4^+^ T cells, effector memory CD4^+^ T cells, regulatory T cells, Naïve CD8^+^ T cells, effector memory CD8^+^ T cells, GZMK^+^ T cells, γδ T cells, NK T cells, and mucosal‐associated invariant T cells (Figure S2a–c). As shown in Figure S2g, mice had more naïve CD8^+^ T cells, but less mucosal‐associated invariant T cells in their splenic immune system compared with long‐lived species. B cells were subdivided into six subpopulations, namely naïve B cells, transitional B cells, marginal zone B cells, follicular B cells, memory B cells, and plasma B cells (Figure S2d–f). Long‐lived species had a significantly higher proportion of naïve B cells than mice, whereas mice had a higher proportion of follicular B cells (Figure S2h).

### 3.3 Iron‐related genes exhibit prominent anti‐immunosenescence feature

Next, we attempted to identity candidate anti‐immunosenescence genes by comparative transcriptomic analysis of long‐lived versus short‐lived mammals. To reduce false positives, candidate genes should meet two criteria. (i) They should exhibit higher expression levels in all these three long‐lived species compared with mouse. Because if a certain gene is more highly expressed in only one long‐lived species, it may be due to species‐specific difference. However, if a certain gene exhibits higher expression level in all the three distantly related species: bat (chiroptera), NMR (rodentia), and human (primates) than mouse (rodentia), it is more likely to involve in the common phenotype of these three long‐lived species, namely, longevity, rather than species difference. (ii) The expression levels of candidate genes should significantly decrease during aging.

**FIGURE 2:**
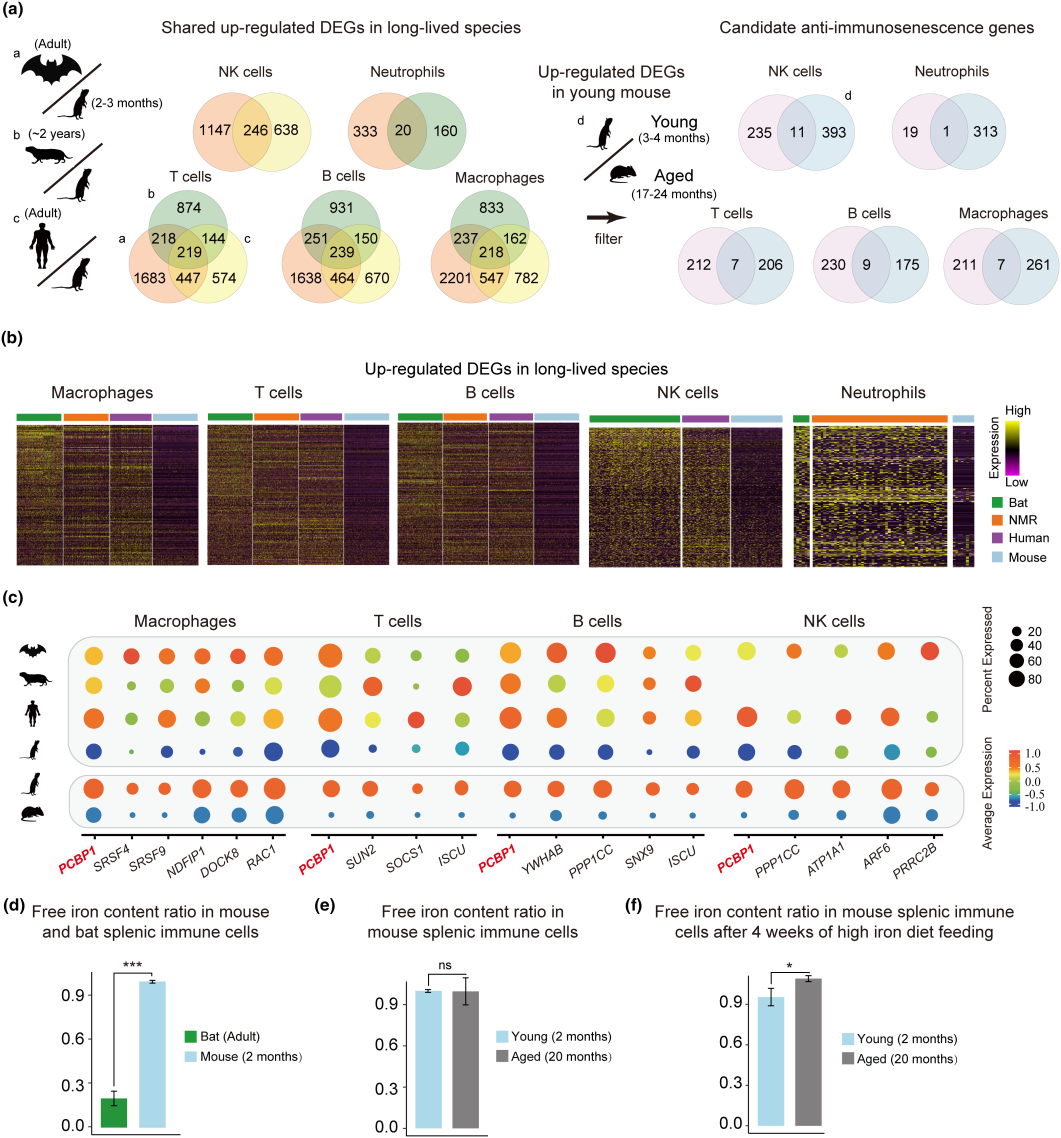
Identification of candidate anti‐immunosenescence genes. (a) Procedures for screening anti‐immunosenescence genes. First, comparison between long‐lived species (bat, NMR and human) and mouse was performed, and the shared upregulated DEGs by all these three long‐lived species in different cell types were shown in venn plots (left). Then, comparison between young and aged mouse was performed to obtain the upregulated DEGs in young mouse (middle), and after filtering, the candidate anti‐immunosenescence genes were finally identified (right). (b) Heatmaps showing the upregulated DEGs in long‐lived species (bat, NMR, and human) compared with mouse. (c) Dotplots showing expression patterns of the anti‐immunosenescence genes in macrophages, T cells, B cells, and NK cells. Comparisons were made across the four species (up), and between the young and aged mouse (bottom). (d) Free iron content ratio in mouse and bat splenic immune cells measured by LIP assay. Three C57BL/6J mice and four myotis bats were used. *** indicates *p* < 0.001 (Student's *t* test). (e) Free iron content ratio in mouse splenic immune cells measured by LIP assay. Three young (2 months old) and three aged (20 months old) C57BL/6J mice were used. ns indicates nonsignificant (Student's *t* test). (f) Free iron content ratio in mouse splenic immune cells after 4 weeks of high iron diet feeding measured by LIP assay. Two young (2 months old) and two (20 months old) C57BL/6J mice were used. * indicates *p* < 0.05 (Student's t test).

Following the above two criteria, we first performed differential expression analysis for every cell type by comparing each of these three long‐lived species with mouse, and only selected upregulated genes that are shared by all these three long‐lived species (Figure 2a,b; Table S3). Then, to check whether their expression levels significantly decrease during aging, we reanalyzed the mouse single‐cell dataset including young and aged splenic immune cells (Mogilenko et al., 2021). We obtained numbers of genes conforming to the above two criteria: higher expression levels in the long‐lived species than in mice and in young mice than in aged mice (Figure 2a; Table S4). Finally, we manually checked the expression patterns of these candidate genes in each cell type among these four species, and identified six genes for macrophages (*PCBP1*, *SRSF4*, *SRSF9*, *NDFIP1*, *DOCK8*, and *RAC1*), four genes for T cells (*PCBP1*, *SUN2*, *SOCS1*, and *ISCU*), five genes for B cells (*PCBP1*, *YWHAB*, *PPP1CC*, *SNX9*, and *ISCU*), five genes for NK cells (*PCBP1*, *PPP1CC*, *ATP1A1*, *ARF6*, and *PRRC2B*) (Figure 2c, Figure S3a,b).

Importantly, three candidate genes showed anti‐immunosenescence feature in more than one cell types: *PCBP1* in all splenic immune cell types, *ISCU* in T and B cells, and *PPP1CC* in B and NK cells, indicating a universal anti‐immunosenescence role (Figure 2c). Besides, *PCBP1*, *ISCU* and *NDFIP1* are also important iron‐related genes. *PCBP1* encoded protein is an important iron chaperone, which could carry and distribute free iron in the form of glutathione linked ferrous (GSH‐Fe II) to other proteins for storage, heme synthesis or implement different biological redox reactions (Ryu et al., 2018). The dysfunction of *PCBP1* may result in extra free iron release and increase lipid peroxidation which induces pathologies such as liver steatosis (Protchenko et al., 2021). *ISCU*, iron–sulfur cluster assembly enzyme, encodes a scaffold protein for the assembly and delivery of iron–sulfur (Fe‐S) clusters, which regulate metabolism and iron homeostasis. Decreased *ISCU* expression can reduce the translation of the iron storage protein FTH1 and lead to iron accumulation in liver cancer (Funauchi et al., 2015). *NDFIP1* mediates ubiquitination and degradation of the ferrous iron transporter DMT1, which is responsible for importing non‐heme iron into cells (Jia et al., 2015). *NDFIP1* also involves in iron homeostasis by ubiquitination and degradation of the iron exporter ferroportin (FPN, encoded by *SLC40A1*) on cell membrane, and *NDFIP1* deficiency increases ferroportin in hepatic macrophages and increase circulating iron (Traeger et al., 2021). Together, these results indicated that iron‐related genes play an important role in counteracting immunosenescence changes.

We also investigated the genes that increase with aging, which may also involve in immunosenescence. We identified genes that exhibit higher expression levels in mice than long‐lived species, and in aged than young mice. In total, 69, 11, 23, 122, and 9 candidate genes were found in T cells, B cells, NK cells, and macrophages, respectively (Figure S3c; Table S4). Next, we filtered genes with low expression level and low fold change, and finally identified three genes for macrophages (*HP*, *CKB*, and *CD79B*), five genes for T cells (*ITM2C*, *CAPNS1*, *SDF4*, *TSPAN13*, and *SMPD13A*), three genes for B cells (*UNC93B1*, *FCGR2B*, and *NAPSA*), seven genes for NK cells (*ITM2C, LTB*, *TCF7*, *SNAP23*, *PLGRKT*, *CD27* and *MFSD10)* (Figure S3d,e). Although these genes may be potential target genes of immunosenescence, it is difficult to distinguish them between mouse species‐specific and immunosenescence features. Therefore, we next mainly focused on candidate genes that were highly expressed in all these three long‐lived species, which theoretically had much lower false positives.

### 3.4 Splenic immune cells of mice have more free iron than those of bats

As iron‐related genes were related to splenic anti‐immunosenescence, we performed iron experiments in splenic immune cells of myotis bats and mice. We measured free iron levels in the form of labile iron pool (LIP) in splenic immune cells by cell cytometry. Compared with bat splenic immune cells, mouse splenic immune cells contained much more free iron (Figure 2d). These results suggested that mouse seems to suffer from higher level of ROS‐induced damage produced by free iron, which may contribute to immunosenescence and undermine longevity (Mangan, 2021).

We also measured free iron levels in splenic immune cells of young and aged mice, but detected no differences (Figure 2e). However, after 4 weeks of high iron diet feeding, we detected higher free iron levels in aged mice than in young mice (Figure 2f). This result indicates that the ability of maintaining iron homeostasis in splenic immune cells of aged mice is reduced, which may promote immunosenescence.

### 3.5 Iron homeostasis is crucial for anti‐immunosenescence

**FIGURE 3:**
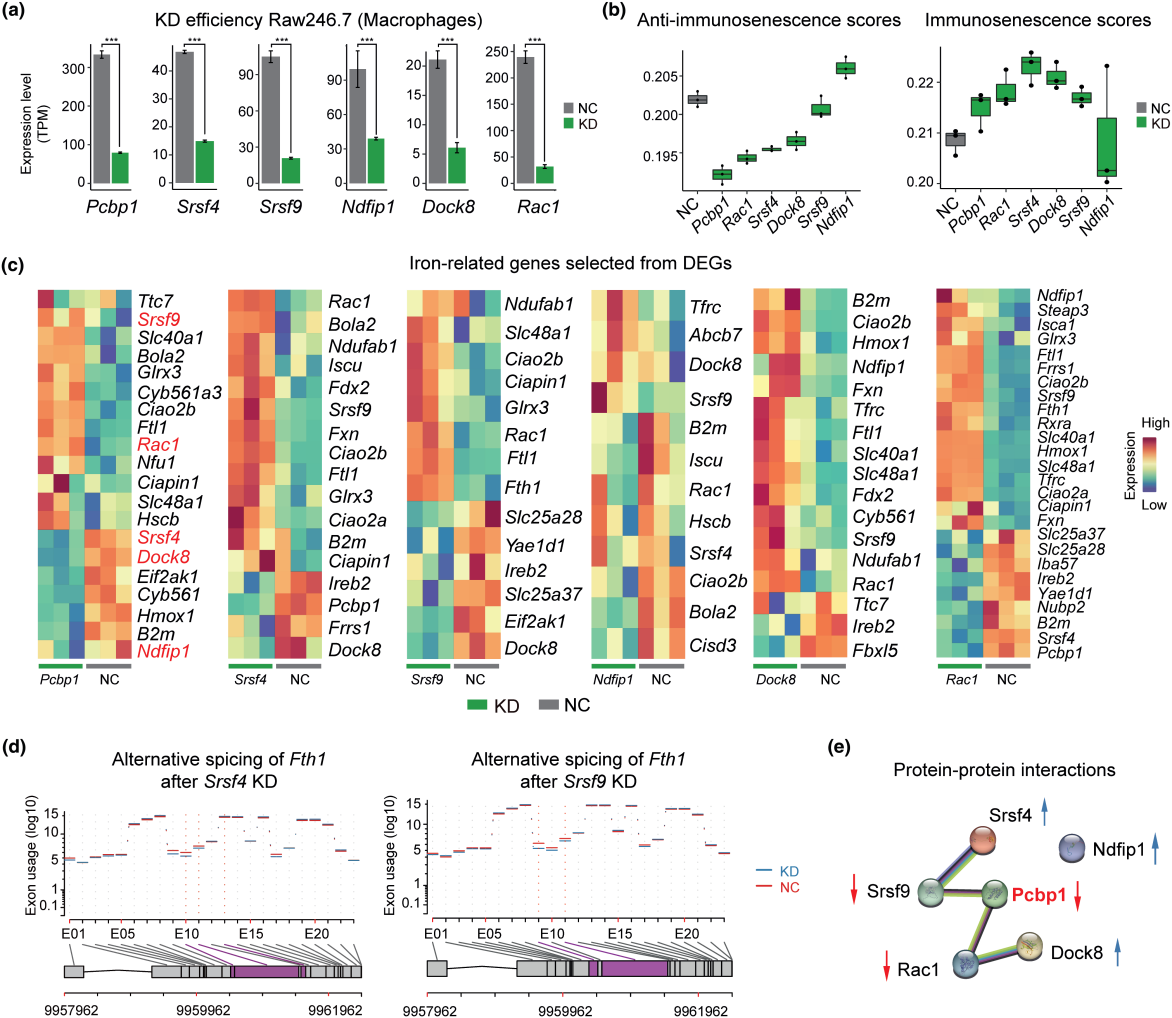
Anti‐immunosenescence gene knockdown results in iron dyshomeostasis in macrophages. (a) siRNA knockdown of *Pcbp1*, *Srsf4*, *Srsf9*, *Ndfip1*, *Dock8*, and *Rac1* in Raw264.7 cell line, non‐target siRNA was used as negative control (NC). Bulk RNA‐seq was preformed 48 h after transfection. Knockdown efficiency was evaluated by comparison between knockdown and NC groups. Three biological replicates were conducted for each group. Data are mean values ± SD. *** indicates *p* < 0.001 (Wald test). (b) The anti‐immunosenescence (left) and immunosenescence (right) scores of anti‐immunosenescence genes knockdown in Raw264.7 cell line, calculated by AuCell using upregulated and downregulated genes in long‐lived species, respectively. (c) Heatmaps of iron‐related genes selected from DEGs between the knockdown samples of anti‐immunosenescence genes and negative control samples in Raw264.7 cell line. (d) Gene location and exon usage change of *Fth1* after *Srsf4* and *Srsf9* knockdown in Raw264.7 cell line. The red lines and blue lines indicate the level of exon usage in the negative control and knockdown groups, respectively. The red dashed lines indicate the affected exons that involved in potential alternative splicing, labeled in purple color, with exon reads count ≥10 at FDR ≤0.05. (e) Protein–protein interaction network of the six anti‐immunosenescence genes identified in splenic macrophages, with *Pcbp1* sitting in the interaction center. Red arrows indicate downregulation and blue arrows indicate upregulation after *Pcbp1* knockdown in Raw264.7 cell line, related to Figure 3c.

To explore the important roles of these anti‐immunosenescence genes in immune cells, we took macrophage as an example and performed siRNA perturbation experiments in mouse macrophage cell line, Raw264.7. Six anti‐immunosenescence genes identified in splenic macrophages were knocked down separately, namely, *Pcbp1*, *Srsf4*, *Srsf9*, *Ndfip1*, *Dock8*, and *Rac1*. As shown in Figure 3a, the knockdown efficiency was pretty high, indicating the success of siRNA experiments. Next, we profiled the transcriptomes of these samples, and calculated the anti‐immunosenescence and immunosenescence scores using upregulated and downregulated genes identified in splenic macrophages of long‐lived species compared with mouse, respectively. Compared with negative control samples, nearly all siRNA experiment samples obtained higher immunosenescence scores but lower anti‐immunosenescence scores, except *Ndfip1* (Figure 3b). These results suggested that the knockdown of these anti‐immunosenescence genes would drive cellular state transition from long‐lived mammal mode to short‐lived mammal mode.

Among these six anti‐immunosenescence genes, *Pcbp1* and *Ndfip1* are crucial to cellular iron homeostasis (Jia et al., 2015; Protchenko et al., 2021). Indeed, *Pcbp1* knockdown in Raw264.7 significantly downregulated iron metabolism‐related genes, such as heme oxidation (*Hmox1*), hemoglobin synthesis (*Eif2ak1*), and iron overload (*B2m*); and also upregulated genes related to iron storage (*Ftl1*, ferritin light polypeptide 1), iron reduction (*Cyb561a3*), iron–sulfur cluster assembly (*Ciao2b* and *Glrx3* and *Bola2*), cellular iron export (*Slc40a1*) (Figures 3c and S4a; Tables S5 and S6). The Fe‐GSH‐PCBP1 complex delivers iron to the Glrx3‐BolA2 complex for cytosolic iron–sulfur [2Fe‐2S] cluster assembly (Patel et al., 2019). The knockdown of *Ndfip1* increased the expression of *Tfrc* and *Abcb7* (Figure 3c). *Tfrc* is the cellular iron gate, controlling the uptake of circulating transferrin‐bound iron to cells (Fillebeen et al., 2019). *Abcb7* is involved in the transfer of iron from mitochondria to the cytoplasm and the maturation of cytoplasmic iron sulfur (Fe/S) proteins (Lehrke et al., 2021).

Unexpectedly, although other four genes (i.e., *Srsf4*, *Srsf9*, *Dock8*, and *Rac1*) were not previously reported to be iron related, their knockdown also severely affected the iron homeostasis in the macrophage cell line (Figure 3c). For example, the knockdown of *Dock8* increased the expression of *B2m*, *Cyb561*, *Fxn*, *Ftl1*, *Hmox1*, *Tfrc*, and *Slc40a1*; the knockdown of *Rac1* increased the expression of genes related to iron ion homeostasis including *Fxn*, *Fth1*, *Ftl1*, *Hmox1*, *Frrs1*, *Tfrc*, *Glrx3*, *Slc40a1*, *Steap3*, *Slc48a*, and iron–sulfur cluster assembly including *Fxn, Glrx3, Ciao2a, Ciao2b, Isca1, Ciapin1*. Also, *Slc25a37* and *Slc25a28* were downregulated in the *Rac1* knockdown group compared with the control group, and both genes were involved in iron import into the mitochondrion (Seguin et al., 2020). It was worth noting that genes related to iron–sulfur cluster assembly were all upregulated in the *Srsf4* and *Srsf9* knockdown groups, including *Glrx3, Ciao2b, Ndufab1*, and *Ciapin1*. Meanwhile, as S*rsf4* and *Srsf9* are crucial in alternative splice site selection during pre‐mRNA splicing (Jeong, 2017), we wondered whether they also played important roles in the alternative splice of iron‐related genes. As shown in Figure S4b,c, the knockdown of *Srsf4* and *Srsf9* indeed affected alternative splicing of some iron homeostasis‐related genes. For example, they both affected *Fth1* exon usage: in the *Srsf4* knockdown group, the tenth, eleventh and thirteenth exon were affected, while in the *Srsf9* knockdown group, the ninth and eleventh exon were affected (Figure 3d).

Importantly, we also detected regulatory relationship among these anti‐immunosenescence genes. We found that *Pcbp1* sat in the center and interacted with other five anti‐immunosenescence genes: the knockdown of *Pcbp1* resulted in the downregulation of *Ndfip1*, *Srsf4* and *Dock8*, and upregulation of *Srsf9* and *Rac1* (Figure 3c). Besides, the protein–protein interaction network also supported their close relationship (Figure 3e). To sum up, all these six identified anti‐immunosenescence genes in splenic macrophages are related to cellular iron homeostasis, and the perturbation of any of them would break the balance of cellular iron trafficking. Thus, their differential expressions between long‐lived and short‐lived mammals, and between young and aged mice, highlight the importance of iron homeostasis in splenic anti‐immunosenescence.

### 3.6 *PCBP1* perturbation results in immunosenescence and iron dyshomeostasis

**FIGURE 4:**
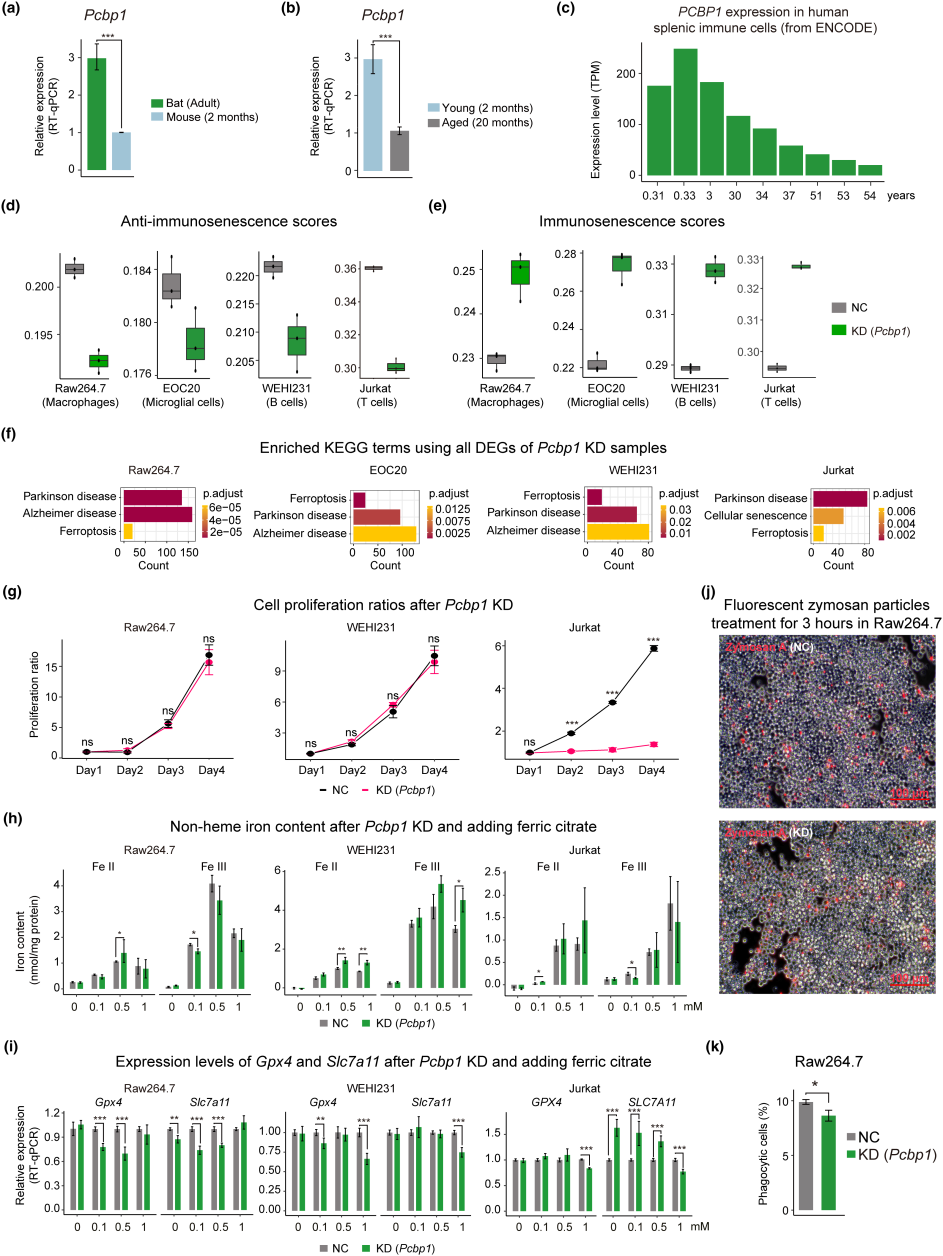
The important role of *PCBP1* in anti‐immunosenescence. (a‐b) The expression levels of *PCBP1* in splenic immune cells evaluated by RT‐qPCR compared between mouse and bat (b), young and aged mice (b). Four biological replicates, each with three technical repeats, were conducted in each sample. Data are mean values ± SD. *** indicates *p* < 0.001 (Student's *t* test). (c) Bar plot showing the change of *PCBP1* expression in human splenic immune cells. Data were downloaded from Encode. (d‐e) The anti‐immunosenescence scores (d) and immunoscenescence scores (e) of *Pcbp1* knockdown in Raw264.7, EOC20, WEHI231, and Jurkat cell lines, calculated by AuCell using the genes upregulated and downregulated in long‐lived species, respectively. (f) Enriched KEGG terms using all DEGs from *Pcbp1* knockdown in Raw264.7, EOC20, WEHI231, and Jurkat cell lines (right), respectively. *p*‐adjusted value <0.05. (g) Cell proliferation ratios after shRNA knockdown of *Pcbp1* in Raw264.7, WEHI23,1 and Jurkat cell lines. (h) Non‐heme iron contents of cells after shRNA knockdown of *Pcbp1* and treatment with different concentrations of ferric citrate. (i) Gene expression quantified by RT‐qPCR in cells after knockdown of *Pcbp1* and treatment with different concentrations of ferric citrate. Four biological replicates, each with three technical repeats, were conducted in each sample. Data are mean values ± SD. * indicates <0.05, ** indicates *p* < 0.01 (Student's *t* test). (j and k) Phagocytosis in *Pcbp1* knockdown Raw264.7 cells after fluorescent zymosan particles treatment for 3 h. Phagocytic cells visualization (j) and calculation by flow cytometry. Three technical repeats were conducted in each sample. Data are mean values ± SD. * indicates <0.05, ns indicates nonsignificant (Student's *t* test).

As *PCBP1* was upregulated in all splenic immune cell types of long‐lived species compared with mouse, we next mainly focused on its role in anti‐immunosenescence. To validate the accuracy of scRNA‐seq results, we performed qPCR experiments for *PCBP1* in bat and mouse splenic immune cells, and also in young and aged mouse splenic immune cells. qPCR results showed higher expression of *PCBP1* in bats than mice, and in young than aged mice, which were consistent with scRNA‐seq results (Figure 4a,b). We also explored the Encode dataset and detected similar expression pattern in human splenic immune cells: the expression level of *PCBP1* dramatically decreased during aging in human (Figure 4c). These results indicate that *PCBP1* function is conserved between human and mouse splenic immune systems.

We then knocked down *PCBP1* in multiple cell lines to verify its effect on anti‐immunosenescence (Figure S5a–d). Consistent with mouse macrophage cell line (Raw246.7), *Pcbp1* knockdown in mouse microglial and B cell lines (EOC20 and WEHI‐231), and human T cell line (Jurkat) also resulted in higher immunosenescence scores but lower anti‐immunosenescence scores (Figure 4d,e). Moreover, KEGG analysis showed that *Pcbp1* downregulation in these immune cell lines all enriched the terms of aging‐related neurodegenerative diseases and ferroptosis, which again confirmed the critical role of *Pcbp1* in anti‐immunosenescence and iron homeostasis (Figure 4f; Tables S7 and S8).

Besides, we also tested the cell viability, changes in iron levels, ferroptosis, and related immune function in immune cell lines with *Pcbp1* knockdown. As shown in Figure 4g, the cell viability of T cells was largely decreased after knockdown, while there were no significant differences in the cell viability of macrophages and B cells. We measured non‐heme iron in immune cell lines by colorimetric assay, and also tested iron tolerance by adding ferric citrate to the medium for 96 h of culture. Both ferrous and ferric iron levels exhibited significant differences between *Pcbp1* knockdown and negative control groups, though the iron patterns varied across different immune cell lines (Figure 4h). Meanwhile, we also performed qPCR experiments for *Gpx4* and *Slc7a11*, whose decreases imply ferroptosis. These two genes exhibited lower expression levels in macrophages of *Pcbp1* knockdown group than negative control group when adding 0.1 mM ferric citrate, while in B and T cells, similar patterns appeared until ferric citrate level reached 1 mM (Figure 4i). Last but not least, after *Pcbp1* knockdown, we detected reduced phagocytosis ability of macrophages (Figures 4j,k and S5e), and immune function‐related genes were significantly changed (Figure S5b). Together, these results further suggest that *PCBP1* perturbation would lead to immunosenescence and iron dyshomeostasis in immune cells.

### 3.7 *PCBP1* is also downregulated during aging in hepatic immune cells

**FIGURE 5:**
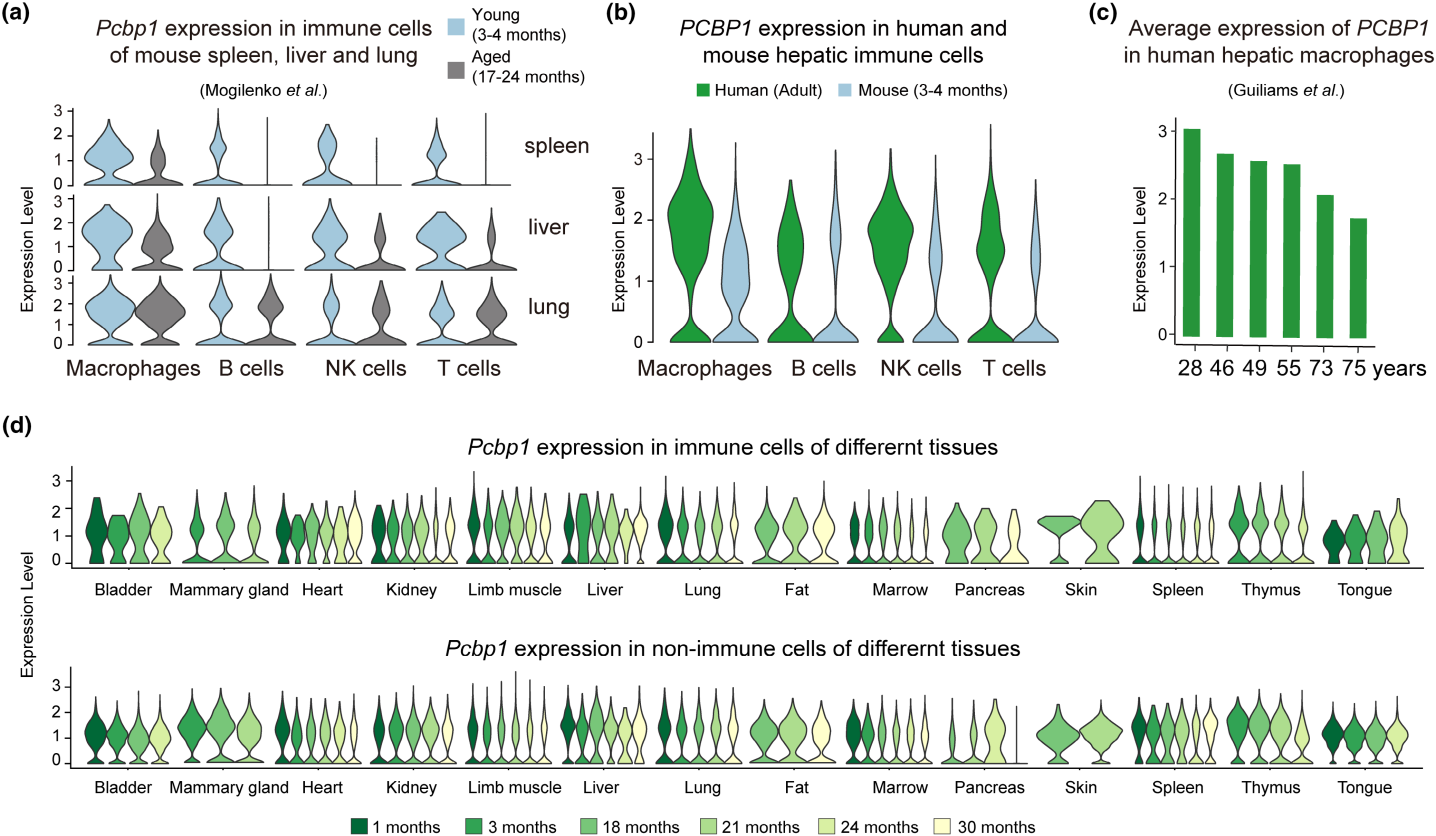
The expression pattern of *PCBP1* in different tissues. (a) Violin plots displaying *Pcbp1* expression level in four immune cell types (macrophages, B cells, NK cells, and T cells) from three organs (spleen, liver, and lung) of young and aged mice. (b) Violin plots displaying the expression level of *PCBP1* in four immune cell types from human and mouse livers. (c) Bar plots showing the average expression level of *PCBP1* in human hepatic macrophages changes with age. Data were downloaded from Liver Cell Atlas (https://livercellatlas.org/download.php). (d) Violin plots showing the expression level of *Pcbp1* in immune and non‐immune cells of different mouse tissues. Data were downloaded from *Tabula Muris Senis*.

Because spleen and liver are both crucial for iron recycling, we wondered whether hepatic immune cells also have similar expression pattern of *Pcbp1* to splenic immune cells. As shown in Figure 5a, *Pcbp1* exhibited resembling expression patterns in mouse splenic and hepatic immune cells, with dramatic decrease during aging. As control, there were no expression differences of *Pcbp1* in pulmonary immune cells between young and aged mice. In addition, the expression level of *PCBP1* was also higher in human than in mouse hepatic immune cells, which was consistent with splenic expression pattern (Figure 5b). Moreover, we reanalyzed human hepatic immune single cell data from a previous study (Guilliams et al., 2022), and detected a downregulation of *PCBP1* in human hepatic macrophages during aging (Figure 5c). These results confirm important roles of spleen and liver in iron recycling, and suggest that *PCBP1* function is conserved between human and mouse hepatic immune systems.

To test whether the role of *Pcbp1* is specific to splenic and hepatic immune systems or also applies to other systems, we examined the expression pattern of *Pcbp1* across multiple organs/tissues using mouse aging cell atlas (*Tabula Muris Sen*is) (The Tabula Muris Consortium et al., 2018). We found that *Pcbp1* expression also decreased with aging in immune cells of thymus and pancreas, but there was no similar expression pattern in nonimmune cells (Figure 5d). This suggested that the role of *Pcbp1* may be specific to the immune systems of certain organs/tissues in mice. Overall, the anti‐immunosenescence feature of *PCBP1* in both splenic and hepatic immune cells was conserved between human and mouse, and the decreased expression of *PCBP1* during aging in immune cells of these two major iron recycling organs further demonstrated that iron dyshomeostasis would aggravate immunosenescence.

As *PCBP2‐4* are the paralogues of *PCBP1*, we also explored the expression pattern of *PCBP2‐4* in splenic immune system across these four species, and between young and aged mice. *PCBP3* and *4* are barely expressed in splenic immune cells, while *PCBP2* expression declines in mouse splenic immune cells during aging (Figure S5f). However, *PCBP2* expression was much lower in splenic immune cells of bats than human, NMRs and even mice. Thus, *PCBP2* does not meet our two criteria for screening candidate genes, and we did not further investigate its role in anti‐immunosenescence.

### 3.8 *PCBP1* can be regulated by three transcription regulators

**FIGURE 6:**
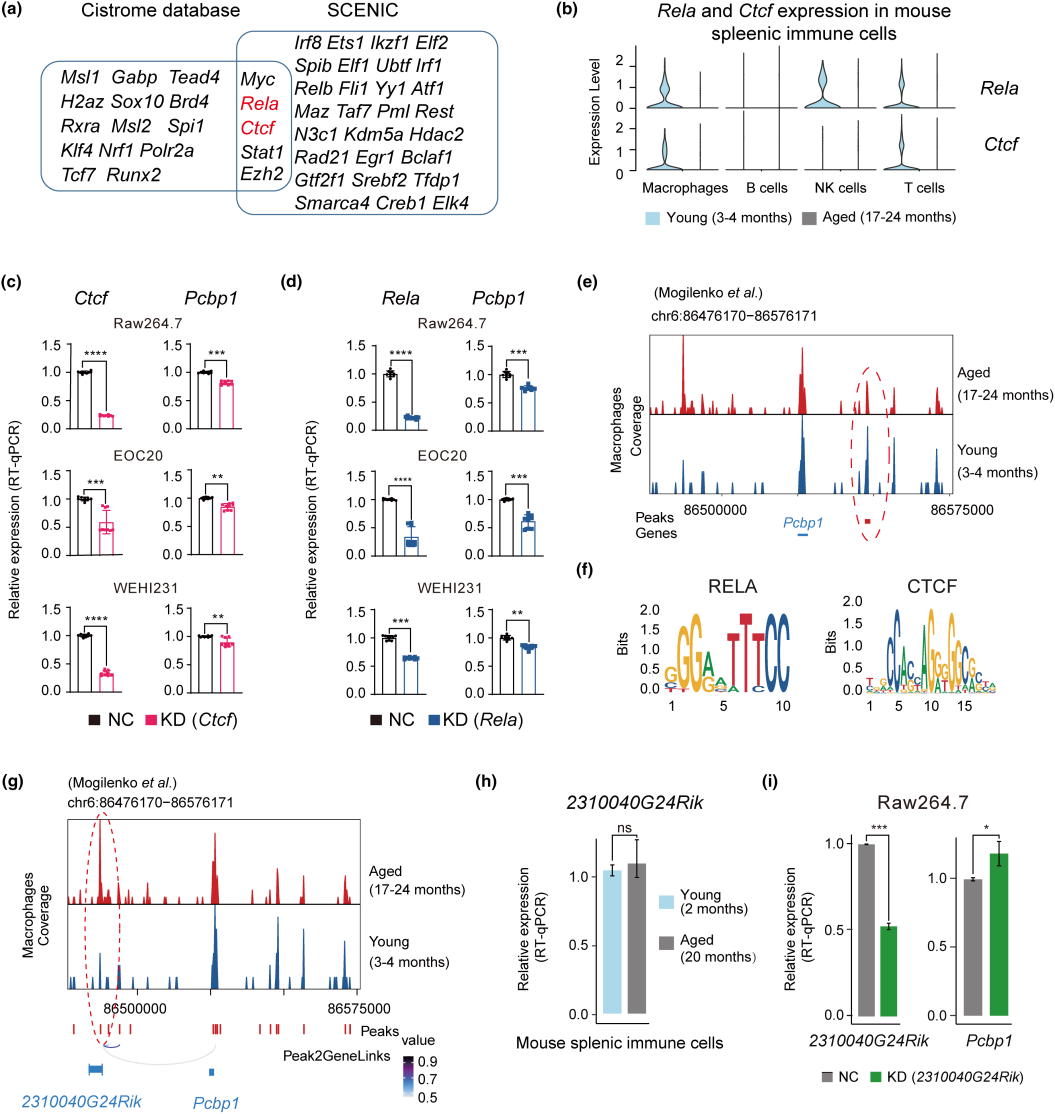
Identification of TFs and lncRNA that regulate *Pcbp1* in mouse splenic immune cells during aging. (a) Intersecting the analysis results of regulatory TFs using Cistrome Toolkit and SCENIC. (b) Violin plots showing the expression levels of *Rela* and *Ctcf* in splenic immune cell types of young and aged mice. (c and d) RT‐qPCR analysis for relative expression of *Pcbp1* after *Ctcf* (c) or *Rela* (d) knockdown in Raw264.7, EOC20, and WEHI231 cell lines. Three biological replicates, each with three technical repeats, were conducted in each sample. **** indicates *p* < 0.0001, *** indicates *p* < 0.001, ** indicates *p* < 0.01 (Student's *t* test). (e) Chromatin accessibility landscape of the *Pcbp1* locus generated by scATAC‐seq of young and aged mouse. The binding region of *Rela* and *Ctcf* was located downstream of *Pcbp1*, circled by dashed red line, which showed higher accessibility in young than aged mice. (f) Binding motifs of RELA and CTCF from JASPAR database. (g) Genome track visualization of *2310040G24Rik* and *Pcbp1* locus. The loops represent the significance of peak‐to‐gene links, indicating the peak near *2310040G24Rik* is associated to *Pcbp1* and its accessibility is higher in aged mouse. (h) RT‐qPCR results showing the expression levels of *2310040G24Rik* in splenic immune cells of young and aged mice. (i) RT‐qPCR analysis for the relative expression of *2310040G24Rik* (left) and *Pcbp1* (right) after *2310040G24Rik* knockdown in Raw264.7 cell line. Four biological replicates, each with three technical repeats, were conducted in each sample. *** indicates *p* < 0.001, * indicates *p* < 0.05 (Student's *t* test).

Now that we have obtained evidence that *PCBP1* is crucial for keeping iron homeostasis and resisting immunosenescence in splenic and hepatic immune cells, we are also eager to know how it is regulated during aging. First, we used Cistrome DB Toolkit to search which transcription factors (TFs) might regulate *Pcbp1* (Zheng et al., 2019). Next, we used SCENIC, a tool to reconstruct gene regulatory networks based on co‐expression and DNA motif analysis, to find TFs that might regulate *Pcbp1* (Aibar et al., 2017). *Myc*, *Rela, Ctcf, Stat1*, and *Ezh2* were identified by intersecting the outputs from these two methods (Figures 6a and S6a,b; Table S9), among which *Rela* and *Ctcf* were downregulated in mouse splenic immune cells during aging, and thus were more likely candidate regulatory TFs of *Pcbp1* (Figure 6b).

To verify the regulatory relationship between *Rela*, *Ctcf*, and *Pcbp1*, we knocked down these two TFs in three immune cell lines, namely, Raw264.7, EOC20, and WEHI231 (Figure 6c,d). As expected, the expression level of *Pcbp1* in the experimental group was significantly lower than that in the control group. In addition, we reanalyzed mouse splenic scATAC‐seq data from a previous study (Mogilenko et al., 2021). Based on the classic marker genes, we annotated four immune cell types including macrophages, NK cells, B cells, and T cells (Figure S6c,d). We found that *Rela* and *Ctcf* can simultaneously bind to the same region, and the accessibility of this region was higher in macrophages of young mice than aged mice (Figure 6e,f), though no difference was detected in other immune cell types (Figure S6e). These results indicate that *Rela* and *Ctcf* can regulate the expression of *Pcbp1* in splenic immune cells, and their decreased expression during aging may result in the downregulation of *Pcbp1* and splenic immunosenescence.

Meanwhile, using the peak2gene function, we also identified a lncRNA, 2310040G24Rik, that could potentially regulate *Pcpb1* at its distal downstream. Moreover, the accessibility of this region is higher in aged than in young mice (Figure 6g). Although the expression level of this lncRNA in mouse splenic immune cells did not significantly increase with aging, the expression of *Pcbp1* was higher in the lncRNA knockdown group than in the control group, indicating its negative regulatory role of *Pcbp1* (Figure 6h,i).

## 4 DISCUSSION

The immune system plays a critical role in identifying and destroying pathogens and nascent malignancies, which is important for organismal homeostasis and longevity (Borgoni et al., 2021). Age‐associated immune decline (i.e., immunosenescence) is concomitant with an age‐dependent increase in chronic diseases in most mammalian species (Borgoni et al., 2021). A commonly used method to study immunosenescence is to make comparison between young and aged model animals (Mogilenko et al., 2021). Although such studies involving in same species with different ages can largely eliminate the effect of false positives, they are also easy to overlook some details that are biologically important but not statistically significant. Another way to tackle immunosenescence is to perform cross‐species comparison between long‐lived and short‐lived species, such as long‐lived naked mole rat or blind mole‐rat versus short‐lived mouse or rat (Hilton et al., 2019; Izraelson et al., 2021). However, it is difficult to distinguish between species‐specific differences and true immunosenescence‐related outcomes. To overcome above problems, we first build the high‐quality reference genome for a long‐lived myotis bat, and then use three long‐lived and distantly related species (i.e., bat (chiroptera), NMR (rodentia), and human (primates)) to perform comparison with the short‐lived mammal, mouse (rodentia), in splenic immune cells. Furthermore, anti‐immunosenescence candidates are filtered by checking their patterns in young and aged mice and/or human. By doing so, these identified molecular clues of anti‐immunosenescence is more likely to involve in the common phenotype of these long‐lived species, namely, longevity, rather than species differences.

One of our noticeable findings is that the B:T cell ratio is significantly higher in mouse than other three long‐lived species, and also higher in aged than young mice, implying its correlation with immunosenescence. This result is in concordance with the declined production and proliferation of naïve T cells, and the deterioration of the supportive stromal cells in lymph node upon aging (Becklund et al., 2016). Lower T cell level in mouse may render the immune system more susceptible to pathogens and age‐related inflammatory factors, hence limiting their lifespan. On the other hand, previous studies found that a new subpopulation of B cells increases with age in mice, which were termed age‐associated B cells (Hao et al., 2011; Rubtsov et al., 2011). These cells can be directly involved in the production of autoantibodies, causing severe autoimmunity and thus promoting aging (Rubtsov et al., 2011). In addition, age‐associated B cells can secrete antibodies in response to toll like receptor stimulation, causing an inflammatory response (Hao et al., 2011). Higher B cell level in mouse may trigger higher autoimmunity and inflammatory response, hence promoting aging.

Furthermore, we identify several iron‐related genes exhibiting prominent anti‐immunosenescence feature: higher expression in long‐lived species and decreased expression during aging. By cross‐species comparison and manually checking the expression patterns, a total of 20 candidate anti‐immunosenescence genes are identified in different splenic immune cell populations. Unexpectedly, in addition to some known iron‐related genes (e.g., *PCBP1*, *ISCU*, and *NDFIP1*), other genes that have not been previously reported to be iron related are also able to regulate iron homeostasis. For example, although there is no evidence that *Srsf4*, *Srsf9*, *Dock8*, and *Rac1* are iron related, knocking down each of them severely affects the iron homeostasis in the macrophage cell line. Besides, we find that bat splenic immune cells contained less free iron but more protein bound iron than mouse splenic immune cells, indicating bat processes stronger ability to control oxidative stress induced by free iron, which may contribute to their longevity (Zeidan et al., 2021). Together, these results highlight the importance of iron homeostasis in splenic anti‐immunosenescence.

Among these anti‐immunosenescence genes, the iron chaperone *PCBP1* best meets the criteria for anti‐immunosenescence. *PCBP1* displays higher expression levels in various immune cell types of the long‐lived species in spleen and liver, both of which are important organs participating in iron storage and recycling, but not in lung. Also, *PCBP1* expression decreases with age in both splenic and hepatic macrophages of human and mouse. Furthermore, the knockdown of *Pcbp1* in multiple cell lines verify its effect on iron metabolism and anti‐immunosenescence. In a recent study, *PCBP1* protects hepatocytes from lipid peroxidation and oxidative‐stress by controlling the labile iron pool (Protchenko et al., 2021). Moreover, *PCBP1* takes part in numerous immune functions. For example, *PCBP1* modulates innate immune response by promoting cGAS binding to viral DNA (Liao et al., 2021). *PCBP1* is also upregulated in activated T cells and prevents the conversion of effector T cells to regulatory T cells (Ansa‐Addo et al., 2020). Therefore, the steady expression of *PCBP1* in splenic immune cells may enable a healthy immune system, and thus is important for anti‐immunosenescence.

Finally, we find *Pcbp1* can be regulated by two TFs, *Rela* and *Ctcf* in mouse splenic immune cells. Knocking down *Rela* or *Ctcf* significantly downregulate the expression of *Pcbp1*, and decreased expression levels of *Rela* and *Ctcf* are detected during aging. A previous study found that the loss of *Rela* would cause ferroptosis in intestinal epithelial cells (Xu et al., 2020). Meanwhile, *Ctcf* deficiency leads to a decrease in cytochrome C and ISCU proteins, which leaded to ROS accumulation and lipid peroxidation (Roy et al., 2018). Iron accumulation and the increase of reactive oxygen species (ROS) produced by lipid peroxidation can trigger ferroptosis (Tang et al., 2021). Consistent with previous studies, we find that *Rela* and *Ctfc* can regulate *Pcbp1* expression, and the knockdown of *Pcbp1* in the cell lines affects the expression of ferroptosis‐related genes. Therefore, we speculate that the decreased expressions of *Rela* and *Ctcf* downregulate the expression of *Pcbp1*, thereby affecting iron homeostasis, which in turn aggravates splenic immunosenescence.

In summary, based on our constructed bat reference genome, we perform a systematical comparison of splenic immune cells between three long‐lived mammals and mouse. We detect a close relationship between B:T cell ratio and immunosenescence, and validate several iron‐related genes exhibiting anti‐immunosenescence feature. Our findings highlight the importance of iron homeostasis in splenic anti‐immunosenescence, and provide novel insights in understanding anti‐immunosenescence and healthy longevity.

## AUTHOR CONTRIBUTIONS

J.D. and M.L. designed and supervised the study, W.H., C.H., J.L., W.D., Q.H., W.C., and L.Z. performed the experiments, Z.H. and H.S. conducted the bioinformatic analyses. J.D., M.L., Z.H., and W.H. wrote the manuscript. All authors reviewed the manuscript.

## CONFLICT OF INTEREST STATEMENT

The authors declare no competing interests.

## Supporting information


Figure S1.

Figure S2.

Figure S3.

Figure S4.

Figure S5.

Figure S6.

Table S1.

Table S2.

Table S3.

Table S4.

Table S5.

Table S6.

Table S7.

Table S8.

Table S9.

Table S10.

## Data Availability

All data are deposited in the Genome Sequence Archive (GSA) database (Accession number: CRA009824). Other resources are available on request.
