# Supplementary figures and images for: Cross‐species comparison illuminates the importance of iron homeostasis for splenic anti‐immunosenescence
Source: Aging Cell. 2023 Sep 8;22(11):e13982. doi: 10.1111/acel.13982 (PMC10652311; doi:10.1111/acel.13982)

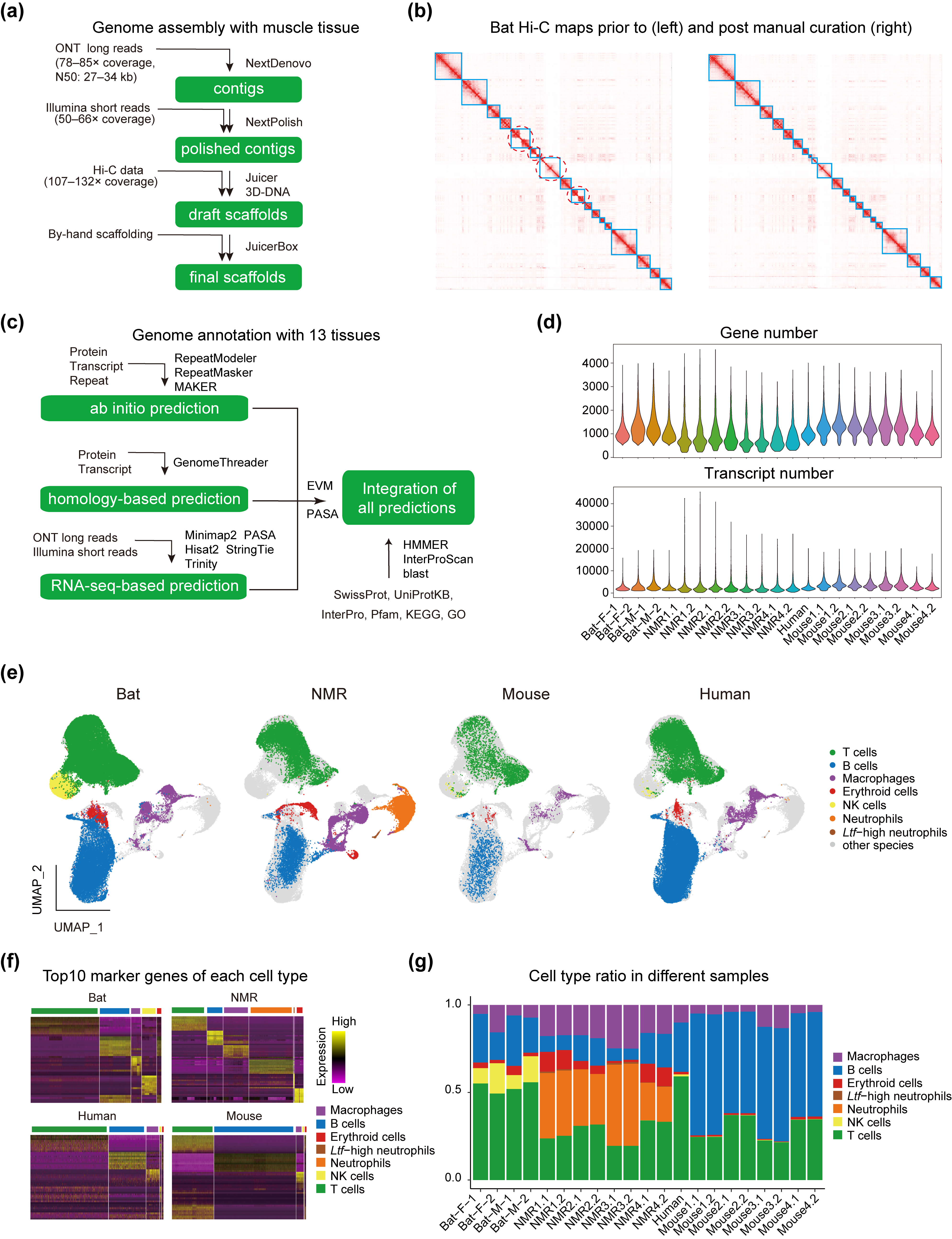

Supplement: Supplementary file 1 — Figure S1. [file ACEL-22-e13982-s016.tif]

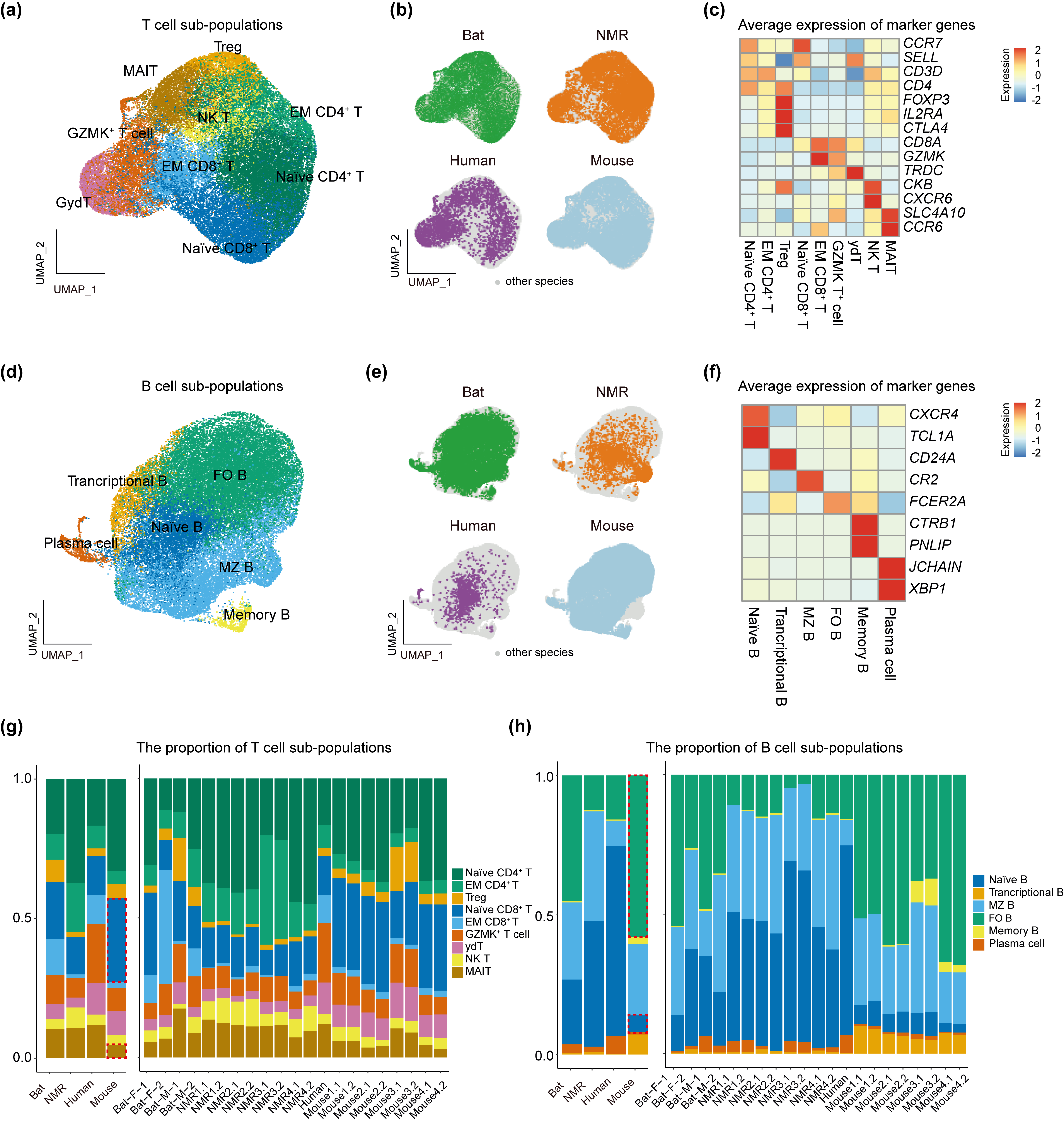

Supplement: Supplementary file 2 — Figure S2. [file ACEL-22-e13982-s004.tif]

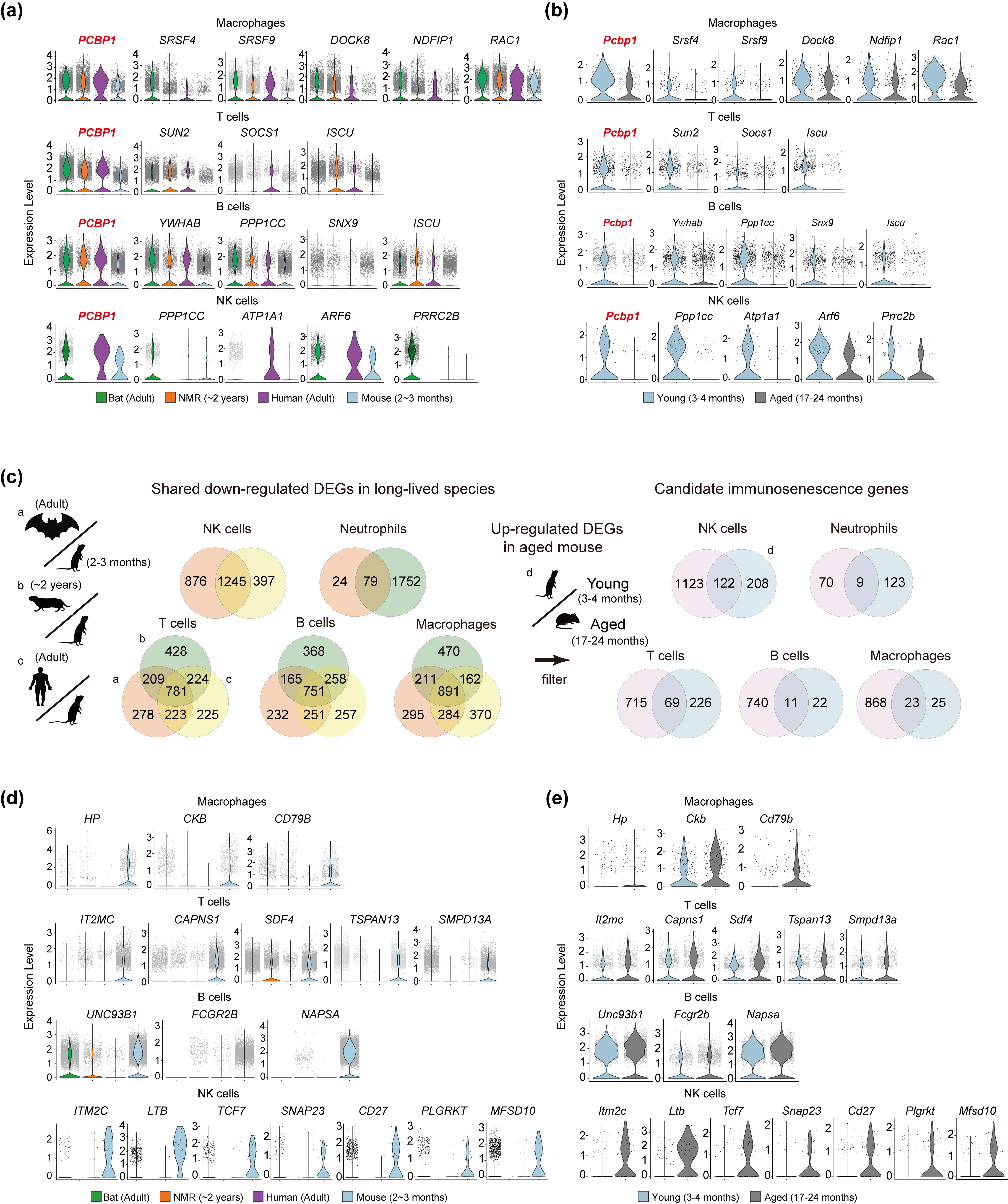

Supplement: Supplementary file 3 — Figure S3. [file ACEL-22-e13982-s008.tif]

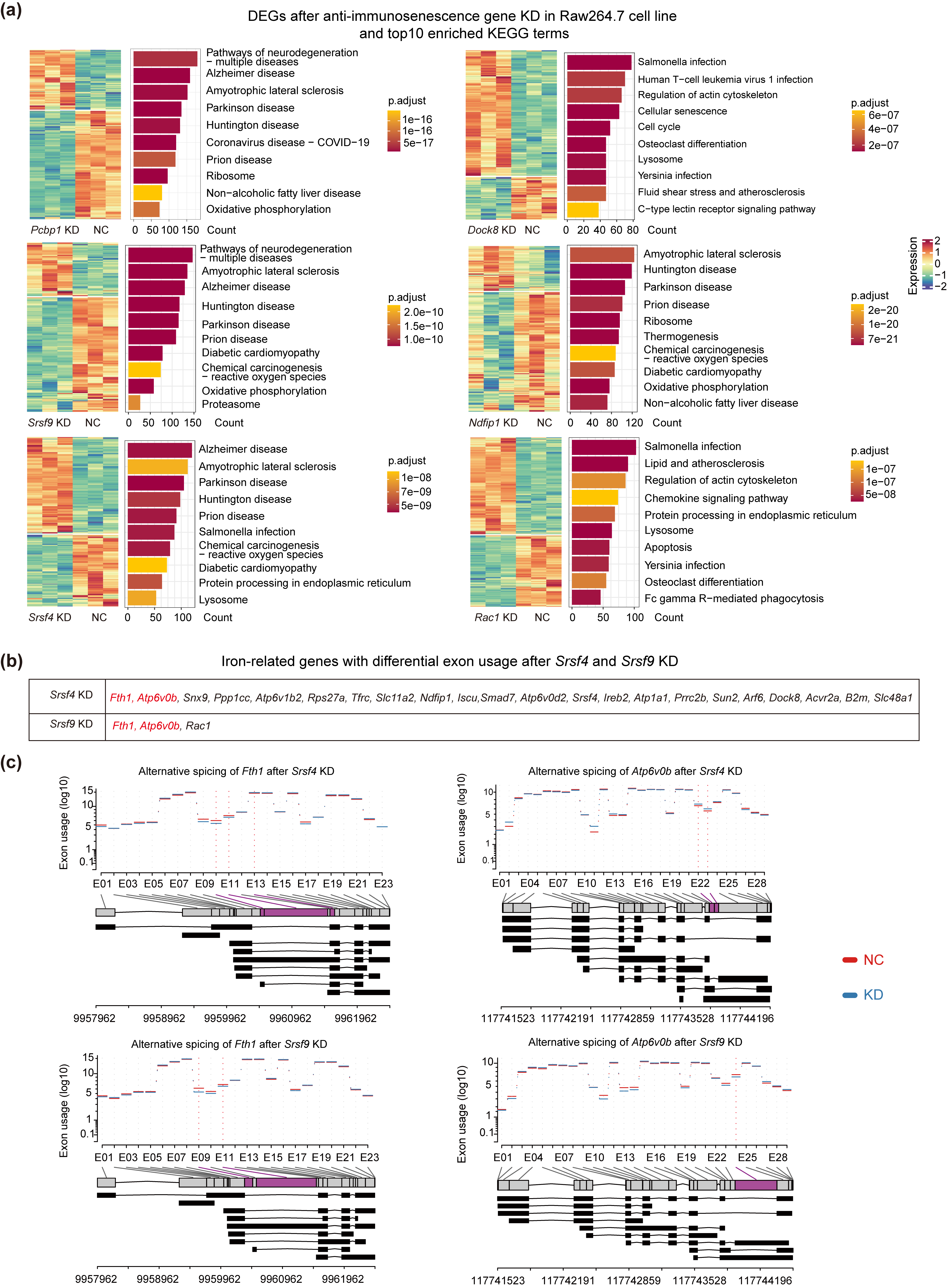

Supplement: Supplementary file 4 — Figure S4. [file ACEL-22-e13982-s011.tif]

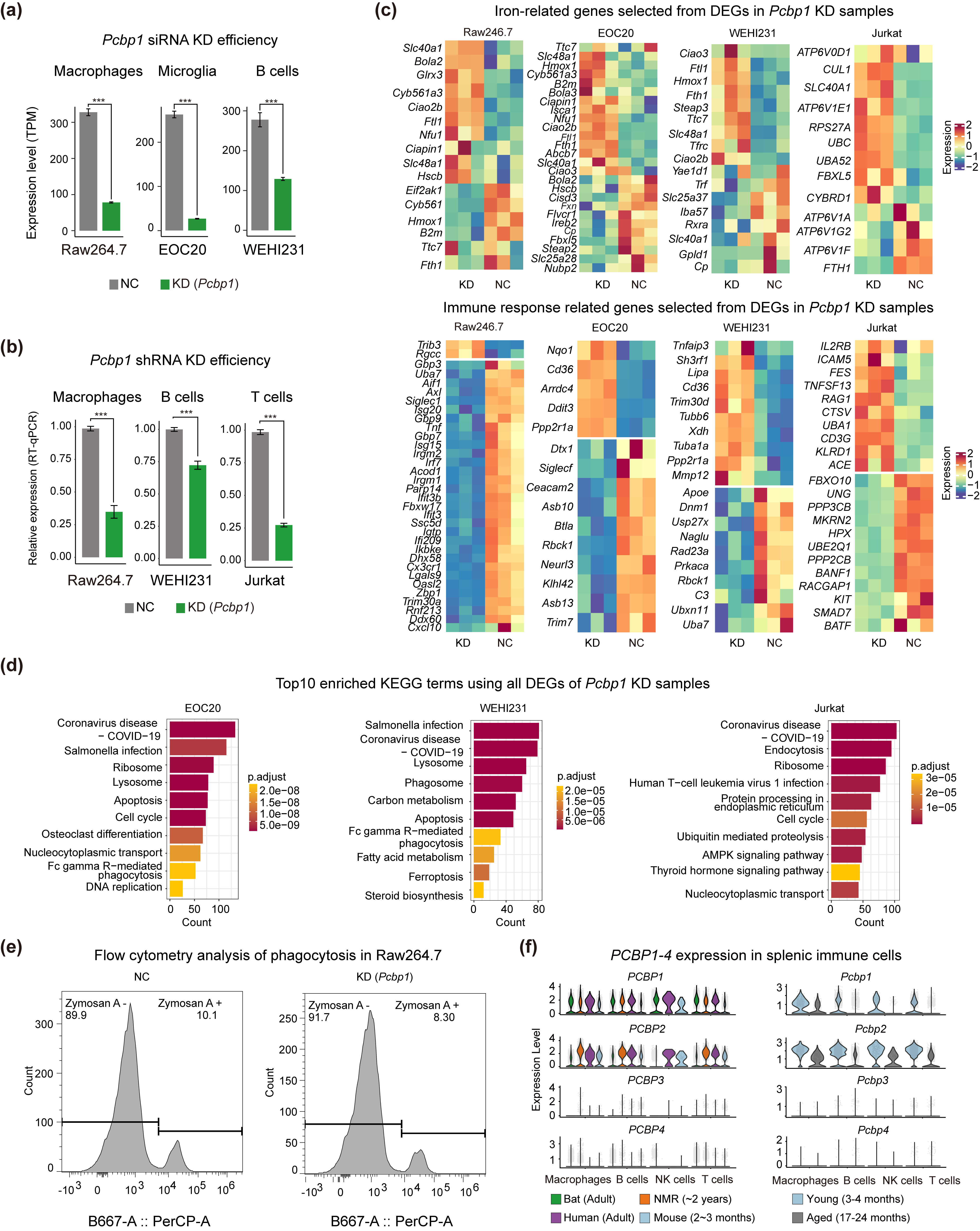

Supplement: Supplementary file 5 — Figure S5. [file ACEL-22-e13982-s013.tif]

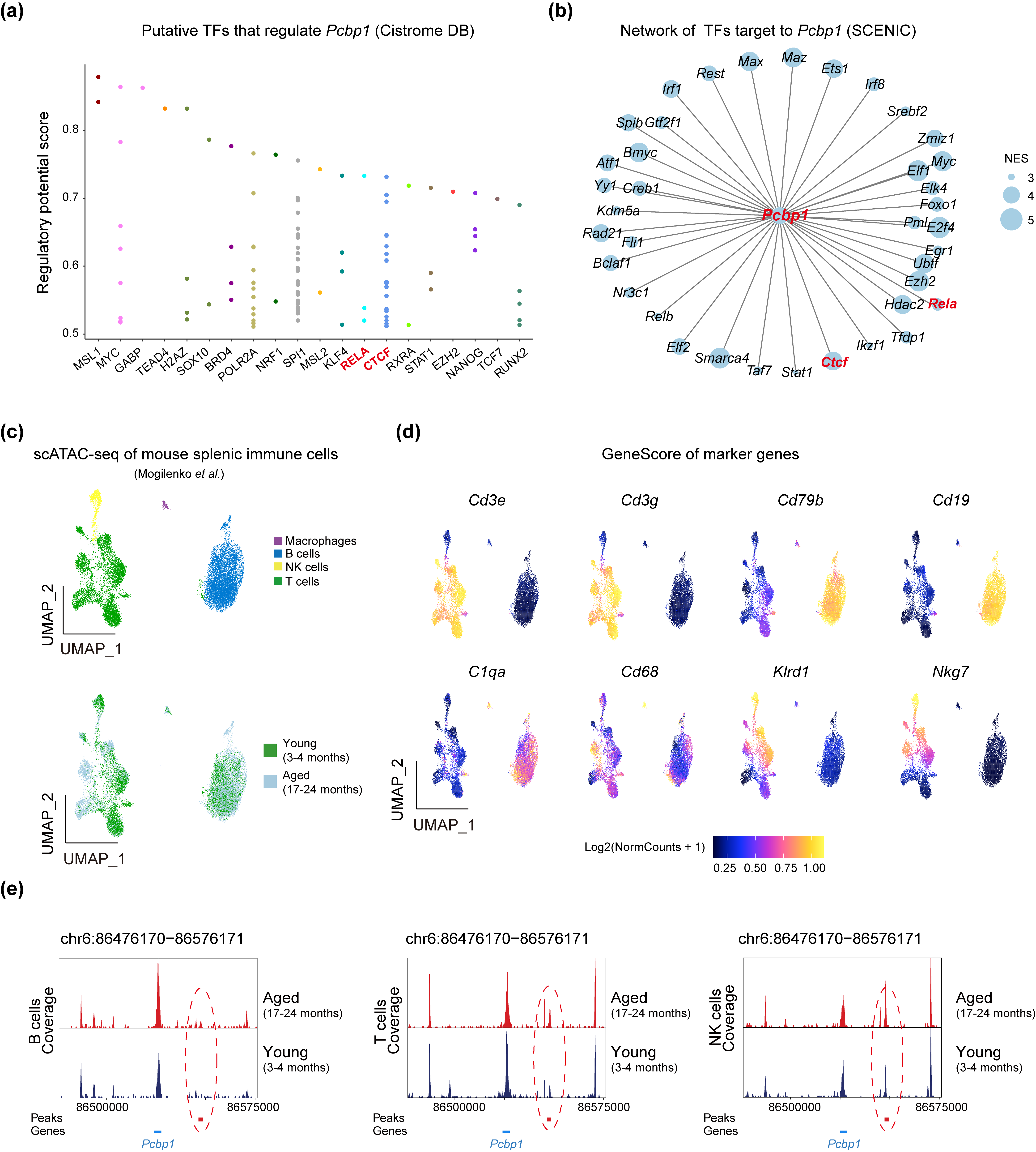

Supplement: Supplementary file 6 — Figure S6. [file ACEL-22-e13982-s006.tif]
